# Retention-Based, Dissociation-Sensitive Pre-Screening of Aptamers Using a Centrifugal Microspin Filter

**DOI:** 10.3390/bios16090510

**Published:** 2026-09-10

**Authors:** Cheeyoon Ahn, Hwayeon Jeong, Kyungjin Jeon, Cheulhee Jung

**Affiliations:** Department of Biotechnology, College of Life Sciences and Biotechnology, Korea University, Seoul 02841, Republic of Korea; k59123@korea.ac.kr (C.A.); cca1987@korea.ac.kr (H.J.); 2021140203@korea.ac.kr (K.J.)

**Keywords:** aptamer, SELEX, kinetic pre-screening, dissociation rate constant, microspin filtration, sandwich detection assay, surface plasmon resonance

## Abstract

Efficient evaluation of aptamer candidates after systematic evolution of ligands by exponential enrichment (SELEX) remains limited by the time and cost required for direct kinetic characterization. We developed a centrifugal microspin filtration (MSF)-based MSF–*k*_off_ assay for retention-based, dissociation-sensitive pre-screening of aptamer candidates. Repeated washing preferentially removes rapidly dissociating aptamers from target-immobilized magnetic beads, and the retained aptamers are quantified by quantitative polymerase chain reaction and expressed as Wash 0-normalized retention (W0-NRT). In a thrombin aptamer panel, W0-NRT showed a strong inverse relationship with SPR-derived *k*_off_. A preliminary analysis of streptavidin aptamers likewise showed a W0-NRT-based ranking consistent with the literature-reported *k*_off_ values, although the two target systems exhibited distinct empirical relationships. In a wash-based thrombin sandwich assay, two affinity-matched sensor aptamers differing 1.76-fold in *k*_off_ were ranked in the same order by post-wash retention as by W0-NRT. The agreement was in rank order rather than in the magnitude of retention. The parallel microspin filter format was also estimated to offer advantages in analysis time and assay-related cost. The MSF–*k*_off_ assay therefore provides a simple post-SELEX approach for prioritizing candidates with higher retention under dissociation-sensitive washing before detailed kinetic characterization.

## 1. Introduction

Aptamers are single-stranded nucleic acid ligands selected to bind specific molecular targets with high affinity and specificity [1] and are used as molecular recognition elements in diagnostics [2,3,4], biosensing [5,6,7], therapeutics [8,9,10], and targeted delivery [11,12,13]. They are obtained by systematic evolution of ligands by exponential enrichment (SELEX), which enriches target-binding sequences from large nucleic acid libraries [14], and the adoption of next-generation sequencing (NGS) now allows the enriched pool to be analyzed on a large scale [15]. Current workflows therefore prioritize candidates by sequence abundance, enrichment across selection rounds, sequence family clustering, and motif analysis [15,16].

Such NGS-based ranking, however, reflects only the extent to which a sequence was enriched during selection, not its target-binding properties or functional performance [17], and enrichment is itself affected by PCR amplification bias and sequence-dependent amplification efficiency, so high abundance does not by itself indicate a functionally superior aptamer [17,18]. Binding properties must therefore be confirmed in a separate post-SELEX evaluation [15], commonly using fluorescence assays, Enzyme-Linked Oligonucleotide Assay (ELONA), or bead-based binding assays, with the equilibrium dissociation constant (*K*_D_) often used as a measure of binding affinity among candidates [19,20].

*K*_D_ alone, however, does not describe the time-dependent nature of an aptamer–target interaction [21]. The association rate constant (*k*_on_) describes how rapidly the complex forms, whereas the dissociation rate constant (*k*_off_) reflects how rapidly the formed complex dissociates [22]. Because *K*_D_ is defined as *k*_off_/*k*_on_, candidates with similar *K*_D_ values can differ substantially in both rate constants [22]. This distinction is particularly relevant in applications requiring sustained target engagement [21,23,24]: in therapeutic targeting and drug delivery, aptamers are continuously challenged by dilution, blood flow, and competing biomolecules, and in biosensing platforms involving washing or flow steps, dissociated aptamers are removed with limited opportunity for rebinding [25,26]. High equilibrium affinity alone therefore may not fully predict practical performance in these settings [27], and a low *k*_off_, with the prolonged target residence time it implies, is an important post-SELEX criterion in its own right [22].

Dissociation kinetics is nevertheless not routinely incorporated into candidate prioritization, because direct kinetic characterization remains experimentally demanding [19,23]. Surface plasmon resonance (SPR) and biolayer interferometry (BLI) can provide quantitative estimates of both rate constants, but require specialized instrumentation, surface immobilization, concentration-series measurements, kinetic fitting, and assay optimization, making their application to large numbers of candidates time- and cost-intensive [28,29]. Because NGS-based SELEX workflows routinely yield tens to hundreds of candidate sequences [30], only a limited subset is advanced to detailed kinetic characterization, leaving a practical workflow gap between high-throughput candidate discovery and low-throughput kinetic characterization.

Strategies that use dissociation behavior to enrich slowly dissociating binders have been described previously. Oh et al. combined a volume dilution challenge with magnetic concentration and continuous washing in a microfluidic channel, reducing rebinding and imposing a stringent selection condition that favors low off-rates [31], and Evenson et al. implemented flow-based kinetic off-rate selection in a microfluidic enrichment device, using controlled flow rather than an external competitor to make ligand retention depend on binding kinetics [32]. Both approaches were designed to enrich slow-dissociating binders during selection rather than to compare individual candidates after it.

Here we describe a post-SELEX pre-screening assay based on centrifugal microspin filtration, in which repeated washing acts as a dissociation-sensitive challenge. Individual candidates are processed with target-immobilized magnetic beads, and the retained aptamer is quantified by qPCR and normalized to the estimated Wash 0 amount to give Wash 0-normalized retention (W0-NRT). We evaluated the assay using thrombin- and streptavidin-binding aptamer panels with distinct kinetic profiles. W0-NRT was compared with SPR-derived *k*_off_ values for the thrombin aptamers and the literature-reported *k*_off_ values for the streptavidin aptamers. We further examined whether the relative ranking obtained with the MSF–*k*_off_ assay was reflected in a wash-based thrombin sandwich detection assay. We also assessed the estimated analysis time and assay-related cost relative to SPR. Collectively, our results suggest that the MSF–*k*_off_ assay provides a simple and accessible platform for prioritizing candidates that show higher retention under dissociation-sensitive washing before detailed biophysical characterization, thereby helping to address the workflow gap between high-throughput NGS-based candidate discovery and low-throughput kinetic characterization.

## 2. Materials and Methods

### 2.1. Materials

All oligonucleotides used in this study were synthesized by Integrated DNA Technologies, Inc. (Coralville, IA, USA). The sequences of all oligonucleotides are listed in Table S1. Hydrophilic streptavidin magnetic beads (S1421S) and the Monarch^®^ Spin PCR & DNA Cleanup Kit (5 μg; T1130) were purchased from New England Biolabs (Ipswich, MA, USA). Tris-HCl buffer, NaCl, MgCl_2_, KCl, and i-StarTaq™ DNA Polymerase were purchased from iNtRON Biotechnology, Inc. (Seongnam, Republic of Korea). The supplied 10× PCR buffer and dNTP mixture were used for qPCR. CaCl_2_ and bovine serum albumin (BSA) were purchased from Sigma-Aldrich (St. Louis, MO, USA). Urea (HC0568) was purchased from HanLAB Corporation (Cheongju, Republic of Korea), and ethanol (1.00983.2511) was purchased from Merck KGaA (Darmstadt, Germany). Ultrafree^®^ centrifugal filters (UFC30HV00) were purchased from Merck Millipore (Burlington, MA, USA). SYTO™ 82 Orange Fluorescent Nucleic Acid Stain (5 mM solution in DMSO; S11363), ROX Reference Dye (12223012), Gibco™ PBS (10×, pH 7.4; 70011044), Invitrogen™ native human α-thrombin (RP-43100), Invitrogen™ biotinylated native human α-thrombin (RP-43103), and a DynaMag™-2 magnet (12321D) were purchased from Thermo Fisher Scientific (Waltham, MA, USA).

### 2.2. Secondary Structure Prediction

The secondary structures of full-length HD22 and M03 were predicted using the mfold web server at https://www.unafold.org/mfold/applications/dna-folding-form.php (accessed on 20 June 2026), with calculations performed at 25 °C under ionic conditions of 100 mM Na^+^ and 2 mM Mg^2+^ [33,34]. The oligomer salt-correction model was applied, and all other parameters were kept at their default settings.

### 2.3. Surface Plasmon Resonance Analysis

Prior to SPR analysis, the thrombin aptamers were heated at 95 °C for 5 min, cooled to 25 °C at a rate of 0.1 °C s^−1^, and subsequently incubated at 25 °C for 30 min using a T100 Thermal Cycler (Bio-Rad, Hercules, CA, USA). SPR measurements were performed at room temperature using an iMSPR-ProX instrument (icluebio, Ansan, Republic of Korea). Biotinylated native human α-thrombin was immobilized on a NeutrAvidin-coated sensor chip to an immobilization level of approximately 6000 response units (RU). A NeutrAvidin-coated channel without immobilized thrombin was used as the reference channel.

The aptamers were serially diluted to concentrations ranging from 5 to 500 nM in running buffer consisting of 20 mM Tris-HCl (pH 7.5), 100 mM NaCl, 2 mM MgCl_2_, 5 mM KCl, 1 mM CaCl_2_, and 0.02% (*v*/*v*) Tween-20. Each aptamer solution was injected at a flow rate of 30 μL min^−1^ for 180 s, followed by a 180 s dissociation phase at the same flow rate. After each injection, the sensor surface was regenerated with 5 mM NaOH to remove bound aptamers from the immobilized thrombin surface. For each aptamer, one concentration series ranging from 5 to 500 nM was measured.

Reference- and buffer blank-subtracted sensorgrams were analyzed using the built-in analysis software of the iMSPR-ProX instrument (version 1.20.9; icluebio, Ansan, Republic of Korea). Concentration-dependent sensorgrams were globally fitted using a 1:1 Langmuir binding model, and the fit included a bulk-effect (BI) term to compensate for the refractive-index difference between the running buffer and the sample solution. The association rate constant (*k*_on_), dissociation rate constant (*k*_off_), and equilibrium dissociation constant (*K*_D_) were determined from the fitted curves, and the χ^2^ value and fitted *R*_max_ returned by the software were recorded as fit-quality information. A 1:1 Langmuir model was used because a single concentration series with a 180 s dissociation phase does not provide sufficient information to identify the additional parameters of heterogeneous-ligand or two-state models.

### 2.4. Preparation of Aptamers and Thrombin-Immobilized Magnetic Beads

The base reaction buffer, which had the same composition as the SPR running buffer, consisted of 20 mM Tris-HCl (pH 7.5), 100 mM NaCl, 2 mM MgCl_2_, 5 mM KCl, 1 mM CaCl_2_, and 0.02% (*v*/*v*) Tween-20. The assay buffer was prepared by supplementing the base reaction buffer with 0.1% (*w*/*v*) BSA and 0.3 μM poly(dT)_30_. The blocking buffer was prepared by supplementing the base reaction buffer with 1% (*w*/*v*) BSA and 2 μM poly(dT)_30_.

For each aptamer, a 200 μL solution was prepared by mixing 100 μL of 2× concentrated base reaction buffer, 96 μL of distilled water, and 4 μL of 10 nM aptamer, resulting in a final aptamer concentration of 0.2 nM. The aptamer solution was divided into four 50 μL aliquots in PCR tubes. Each aliquot contained 10 fmol of aptamer, corresponding to 6.02 × 10^9^ copies. The aliquots were heated at 95 °C for 5 min, cooled to 25 °C at a rate of 0.1 °C s^−1^, and subsequently incubated at 25 °C for 30 min using a T100 Thermal Cycler. One folded aliquot was reserved as the input control and directly used for qPCR quantification without bead incubation or washing. The remaining aliquots were used for the MSF–*k*_off_ assay.

Hydrophilic streptavidin magnetic beads (115 μL, 4 mg mL^−1^; 460 μg) were mixed with 385 μL of 1× PBS containing 0.02% (*v*/*v*) Tween-20 (PBST) and rotated at 25 °C for 2 min. The beads were collected on a magnetic stand for 2 min, and the supernatant was carefully removed. The beads were resuspended in 500 μL of PBST by pipetting and washed three times in total.

For thrombin aptamer assays, a 4.6 μM thrombin working solution was prepared by mixing 23 μL of 2× PBST, 17 μL of distilled water, and 6 μL of 35.4 μM biotinylated native human α-thrombin. The washed beads were resuspended in 30 μL of 1× PBST and mixed with 20 μL of the thrombin working solution, resulting in a total volume of 50 μL containing 92 pmol of thrombin at a final concentration of 1.84 μM. The suspension was rotated at 25 °C for 1 h to immobilize thrombin on the bead surface. For streptavidin aptamer assays, this protein immobilization step was omitted because the streptavidin coating of the beads served directly as the target.

The beads were collected on a magnetic stand for 2 min and washed three times with 500 μL of assay buffer. The beads were subsequently resuspended in 500 μL of blocking buffer and rotated at 25 °C for 1 h. After blocking, the beads were collected on the magnetic stand for 2 min and washed three times with 500 μL of assay buffer. Finally, the beads were resuspended in 250 μL of assay buffer.

### 2.5. MSF–k_off_ Assay

For each assay condition, three microspin filter units were processed in parallel and treated as parallel process replicates. Each filter unit was conditioned with 500 μL of assay buffer by centrifugation at 12,000× *g* for 1 min. The filter units were then transferred to clean collection tubes.

For optimization of the assay conditions using HD22 and M03, the numbers of sample passages and wash cycles were varied. For sample-passage optimization, the aptamer solution was passed through the bead-containing filter unit 1, 3, 10, or 20 times, followed by immediate elution without washing. For wash-cycle optimization, 10 sample passages were followed by 0, 1, 3, 10, or 20 wash cycles before elution. Unless otherwise specified, subsequent MSF–*k*_off_ assays were performed using 10 sample passages and 10 wash cycles.

A 50 μL aliquot of the prepared target bead suspension was loaded onto each filter unit, followed by 50 μL of the folded aptamer solution containing 10 fmol of aptamer. The bead suspension and aptamer solution were mixed thoroughly by pipetting and centrifuged at 12,000× *g* for 1 min. The collected flow-through was reapplied to the same filter unit and centrifuged under the same conditions nine additional times, resulting in a total of 10 sample passages. The flow-through obtained after the tenth sample passage was collected as the FT fraction.

Following the repeated sample passages, each filter unit was washed 10 times with 400 μL of fresh assay buffer. Each wash was performed by centrifugation at 12,000× *g* for 1 min. Bound aptamers were subsequently eluted by adding 100 μL of 8 M urea to the filter unit and centrifuging at 12,000× *g* for 1 min.

The resulting 100 μL eluate was mixed with 200 μL of Monarch Buffer BZ and 600 μL of ethanol (>95%) by pipetting without vortexing. The entire mixture was loaded onto a Monarch^®^ Spin PCR & DNA Cleanup column in two 450 μL aliquots, with each loading followed by centrifugation at 16,000× *g* for 1 min. The flow-through was discarded after each centrifugation.

The cleanup column was washed twice with 500 μL of Monarch Buffer WZ by centrifugation at 16,000× *g* for 1 min. After an additional centrifugation at 16,000× *g* for 1 min to remove residual wash buffer, the column was transferred to a fresh 1.5 mL microtube. Nuclease-free water preheated to 50 °C (50 μL) was applied directly to the center of the column matrix. After incubation for 1 min, the purified aptamer was recovered by centrifugation at 16,000× *g* for 1 min in a final volume of 50 μL.

The two washing formats were compared using HD22 and M03. For both formats, folded aptamer and thrombin-immobilized beads were incubated on a rotator at room temperature for 1 h under the same binding conditions. The two formats differed only in the washing step. For W0 measurements, samples were eluted immediately after the binding incubation without washing. For W10 measurements, samples were washed 10 times with 400 μL of assay buffer. In the filter arm, each wash was performed by centrifugation through the microspin filter unit, whereas in the magnetic arm, each wash consisted of rotation at room temperature for 1 min, followed by collection of the beads on a magnetic stand and removal of the supernatant. Elution, DNA cleanup, and qPCR quantification were performed identically for all samples. Each washing format was evaluated using three parallel process replicates.

To test the assumption underlying the estimated Wash 0 amount, a no-wash control was performed for M09, HD22, and AYA1809002. These aptamers were processed exactly as described above through the ten sample passages, after which the flow-through was collected and the beads were eluted immediately without any wash cycle. The eluate was purified and quantified as described, so that the amount recovered from the beads could be compared directly with the amount estimated as the difference between the input and the flow-through.

To characterize aptamer release during successive washes, M09, HD22, and AYA1809002 were assayed under the standard conditions, with each of the ten wash fractions collected separately. The flow-through from the sample-passage step and the final eluate were also collected. Each fraction was purified and quantified by qPCR, and the recovered amount was expressed as a percentage of the corresponding input amount.

To assess day-to-day reproducibility, the complete MSF–*k*_off_ workflow was repeated on two separate experimental days for M09, HD22, and AYA1809002. On each day, aptamer folding, bead preparation, thrombin immobilization, and the qPCR standard curve were prepared independently, and three microspin filter units were processed as parallel process replicates.

As a composition-matched control, a scrambled version of HD22 was prepared by randomly rearranging the aptamer-derived region while retaining the 5′ and 3′ constant regions and the nucleotide composition of the original aptamer-derived sequence. The scrambled HD22 was folded and applied to thrombin-immobilized magnetic beads under the same MSF–*k*_off_ assay conditions used for HD22. The flow-through and final eluate fractions were collected and quantified by qPCR, and the recovery of each fraction was normalized to the input amount. The scrambled HD22 sequence is provided in Table S1.

### 2.6. Quantification of Aptamers by qPCR

The copy numbers of the input, flow-through (FT), and eluate fractions were quantified by quantitative PCR (qPCR) using a StepOnePlus™ Real-Time PCR System (Applied Biosystems, Thermo Fisher Scientific, Waltham, MA, USA). Each 20 μL qPCR reaction contained 11.1 μL of distilled water, 2 μL of 10× PCR buffer, 2 μL of dNTP mixture (2.5 mM each), 1 μL of 50 μM SYTO™ 82, 1 μL each of 10 μM forward and reverse primers, 0.4 μL of 50× ROX reference dye, 0.5 μL of i-StarTaq™ DNA Polymerase, and 1 μL of the sample or standard template.

For thrombin aptamers, the cycling conditions consisted of initial denaturation at 94 °C for 2 min, followed by 40 cycles of denaturation at 94 °C for 20 s, annealing at 60 °C for 10 s, and extension at 72 °C for 30 s. For streptavidin aptamers, the cycling conditions consisted of initial denaturation at 94 °C for 2 min, followed by 50 cycles of denaturation at 94 °C for 20 s, annealing at 52 °C for 30 s, and extension at 72 °C for 30 s. Each fraction from each parallel process replicate was quantified by qPCR in technical triplicate.

For each aptamer, a seven-point standard curve was generated using 10-fold serial dilutions of an aptamer-specific oligonucleotide standard ranging from 100 fmol to 100 zmol per reaction, prepared from the same synthesized oligonucleotide used as the assay input. The primer pairs were common to candidates within the same construct family, whereas the standard curve was prepared separately for each candidate. A no-template control (NTC) was included in each qPCR run. Aptamer copy numbers were calculated by interpolation from the linear regression of the threshold cycle values against the log10-transformed standard amounts. The total copy number in each fraction was calculated by correcting for the dilution factor and the total fraction volume. The primer sequences are provided in Table S1.

The lowest standard point, 100 zmol per reaction, corresponding to approximately 6.0 × 10^4^ copies per reaction, was used as the lower limit of quantification (LLOQ). Because each fraction was recovered in 50 μL after cleanup and 1 μL was used per qPCR reaction, this corresponded to approximately 3.0 × 10^6^ copies per fraction. Values below this limit were reported as <LLOQ and were not used as quantitative values. All input, flow-through, and eluate fractions used for quantitative analysis fell within the standard-curve range. The approximate amounts per qPCR reaction were 200 amol for the input, up to 136 amol for the flow-through fractions, and 12–120 amol for the eluate fractions.

To evaluate the effects of the buffer matrix and oligonucleotide cleanup procedure on qPCR quantification, a recovery test was performed using the same amount of oligonucleotide as that used for the assay input. Equal amounts of oligonucleotide were prepared in distilled water or in 1× base reaction buffer and quantified directly by qPCR. To evaluate recovery through the cleanup step, equal amounts were prepared in distilled water or in 8 M urea, corresponding to the elution condition of the MSF–*k*_off_ assay, subjected to the same oligonucleotide cleanup procedure, and then quantified by qPCR. Recovery for each condition was calculated relative to the distilled-water condition without cleanup, which was set to 100%. This experiment evaluated recovery under the relevant buffer and elution conditions but was not a matrix-spike experiment using actual input, flow-through, or eluate fractions collected from a completed MSF–*k*_off_ assay.

### 2.7. Calculation of MSF–k_off_ Assay Metrics

The results of the MSF–*k*_off_ assay were represented using two normalization metrics. First, input-normalized recovery (I-NRC) was defined as the percentage of aptamer copy number recovered in the final eluate relative to the total aptamer copy number introduced into the assay. Unless otherwise stated, the I-NRC values reported in this study were obtained under the no-wash condition, in which aptamers were eluted immediately after the sample-passage step. I-NRC therefore reflects the fraction of input aptamer recovered after repeated sample passage under the no-wash condition and should not be interpreted as an equilibrium bound fraction.

(1) $$\mathrm{Input}-\mathrm{normalized}\;\mathrm{recovery}\;(\%)=\frac{{CN}_{eluate}}{{CN}_{input}}\times 100$$

To more directly evaluate aptamer retention after repeated washing, Wash 0-normalized retention (W0-NRT) was also calculated. W0-NRT was calculated by normalizing the aptamer copy number recovered in the final eluate after 10 wash cycles to the aptamer copy number estimated to remain on the target-immobilized beads at the end of the repeated sample-passage step. This reference state was defined as Wash 0 and corresponds to the state immediately before the first wash cycle. Because aptamers can undergo both association with and dissociation from the target during sample passage, Wash 0 does not represent the state immediately after the initial contact with the target or a state before dissociation has occurred. The Wash 0 amount was estimated by subtracting the total copy number recovered in the flow-through fraction after repeated sample passage from the input copy number.

(2) $$\mathrm{Wash}\;0\text{-}\mathrm{normalized}\;\mathrm{retention}\;(\%)=\frac{{CN}_{eluate,W10}}{{CN}_{input}-{CN}_{FT}}\times 100$$

Here, CN_input_, CN_FT_, and CN_eluate,W10_ represent the total aptamer copy numbers in the input, the total flow-through fraction after repeated sample passage, and the final eluate after 10 wash cycles, respectively. Accordingly, CN_input_ − CN_FT_ represents the estimated amount of aptamer remaining on the target-immobilized beads at the end of the repeated sample-passage step. W0-NRT was calculated by normalizing the amount of aptamer recovered in the final eluate after 10 wash cycles to this estimated Wash 0 amount and was used as a retention-based metric to reduce the influence of differences in capture during sample passage and to compare retention during washing.

Because CN_input_ − CN_FT_ is an estimate of the amount retained rather than a direct measurement, this estimate was compared with a no-wash control for three aptamers spanning the *k*_off_ range of the thrombin panel (Section 3.2 and Figure S3). W0-NRT was calculated separately for each parallel process replicate using the input, flow-through, and eluate measurements obtained from the same replicate. Variability in the estimated Wash 0 denominator among process replicates was therefore included in the reported SD values. Technical uncertainty associated with the qPCR measurement of individual fractions was not propagated separately into the W0-NRT calculation.

### 2.8. Statistical Analysis

W0-NRT was calculated separately for each parallel process replicate, and the mean W0-NRT value for each aptamer was subsequently ln-transformed for correlation and regression analyses. Relationships between W0-NRT and the SPR-derived equilibrium and kinetic parameters were assessed by Pearson and Spearman correlation. Because *K*_D_ is defined as *k*_off_/*k*_on_, the three parameters are not independent, and partial Pearson correlations were therefore also computed between *k*_off_ and ln-transformed W0-NRT with *K*_D_ or *k*_on_ held constant and, conversely, between *K*_D_ or *k*_on_ and ln-transformed W0-NRT with *k*_off_ held constant. Multivariable linear regression of ln-transformed W0-NRT on *k*_off_ together with *K*_D_, and on *k*_off_ together with *k*_on_, was performed for the same purpose, with variance inflation factors calculated to assess collinearity. A model containing all three parameters was not fitted because their algebraic dependence makes such a model unidentifiable. Pairs of aptamers matched in one binding parameter while differing in others were compared using Welch’s *t* test on the replicate-level W0-NRT values.

The empirical relationship in Equation (6) was fitted by ordinary least squares and, to account for measurement uncertainty in the retention values, also by weighted least squares, with each point weighted by the inverse of the squared standard error of its ln-transformed W0-NRT; both estimates are reported. Uncertainty in *k*_off_ was not incorporated because each aptamer was analyzed by SPR using a single concentration series fitted globally and no replicate-level standard error was available.

For the primary analyses of the relationship between W0-NRT and *k*_off_, the significance of the Spearman correlation was assessed by exhaustive enumeration of all permutations for the six-aptamer streptavidin panel and by permutation tests with 10^6^ resamples for the thrombin panel and its sensitivity analyses. Ninety-five percent confidence intervals for the correlation coefficients were obtained by bootstrap resampling of the aptamers with 10,000 resamples, whereas the confidence interval for the regression coefficient D was the ordinary least-squares interval, and leave-one-out analysis was performed by recalculating each statistic with one aptamer omitted in turn. A sensitivity analysis excluding HD22 and M03, which were used to establish the operating conditions, was performed for the thrombin panel. Correlation coefficients, regression coefficients, confidence intervals, and *p* values are reported for each analysis.

### 2.9. Sandwich Detection Assay

Before the sandwich detection assay, the compatibility of HD1 and M03 for simultaneous thrombin binding was evaluated. Streptavidin magnetic beads were incubated with 18.4 pmol of biotinylated thrombin for 1 h to immobilize the target. The beads were washed three times with 500 μL of assay buffer, with each wash including a 2-min incubation, and subsequently incubated with or without HD1 (200 pmol) for 1 h, followed by the same washing procedure. M03 (10 fmol) was then added and incubated for 1 h. After a final three washes using the same procedure, bound M03 was eluted with 50 μL of 8 M urea by incubation for 2 min at room temperature. The eluate was subjected to oligonucleotide cleanup and quantified by qPCR as described in Section 2.5 and Section 2.6. The amount recovered in the HD1-present condition was normalized to the mean value obtained in the absence of HD1, which was defined as 100%. Three parallel assay replicates were analyzed, with each sample quantified by qPCR in technical triplicate.

For the sandwich detection assay, streptavidin magnetic beads were incubated with 20 pmol of biotinylated HD1 for 1 h and washed three times with 500 μL of assay buffer, with each wash including a 2-min incubation. The HD1-immobilized beads were incubated with thrombin at 0, 0.5, 5, or 50 nM in 50 μL for 1 h, followed by the same washing procedure. HD22 or M03 (5 pmol in 50 μL) was then added as the detection aptamer and incubated for 1 h. For the unwashed condition (W0), the detection aptamer was eluted immediately without further washing; for the washed condition (W10), the beads underwent ten sequential washes with 500 μL of assay buffer, with each wash including a 2-min incubation. For both conditions, the retained detection aptamer was eluted with 50 μL of 8 M urea by incubation for 2 min at room temperature, followed by oligonucleotide cleanup and qPCR quantification as described in Section 2.5 and Section 2.6. Post-wash retention was calculated as W10/W0 × 100 using the corresponding unwashed value at each thrombin concentration. Component-omission controls were performed at 50 nM thrombin by omitting HD1, thrombin, or the detection aptamer from the complete sandwich assay. Each condition was analyzed in three parallel assay replicates, with each sample quantified by qPCR in technical triplicate.

## 3. Results and Discussion

### 3.1. Design and Principle of the MSF–k_off_ Assay

**Figure 1:**
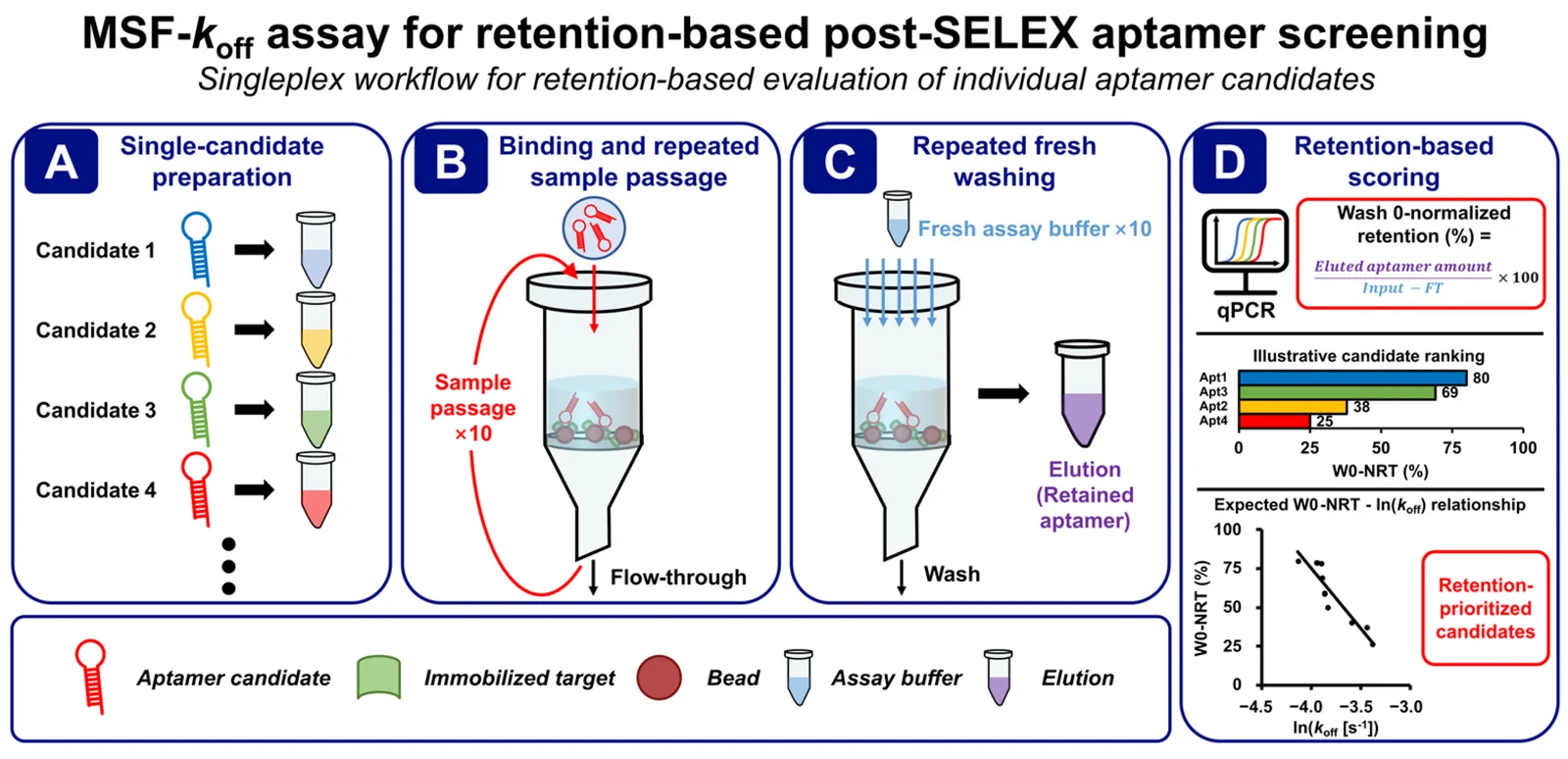
Schematic workflow and principle of the MSF–*k*_off_ assay for retention-based post-SELEX aptamer screening. (**A**) Preparation of individual aptamer candidates for singleplex evaluation. (**B**) Binding of each aptamer to target-bearing magnetic beads through 10 repeated sample passages. (**C**) Elution of retained aptamers after 10 washes with fresh assay buffer. (**D**) qPCR-based calculation of W0-NRT, illustrative candidate ranking, and the expected inverse relationship between retention and *k*_off_. W0-NRT was calculated as the amount of eluted aptamer divided by the difference between the input and flow-through (FT) amounts, multiplied by 100. The values and plot in (**D**) are illustrative examples and do not represent experimental data. W0-NRT indicates Wash 0-normalized retention, and FT indicates the flow-through fraction.

To establish a post-SELEX screening method capable of prioritizing aptamers according to their dissociation behavior, we developed the MSF–*k*_off_ assay, whose overall workflow and retention-based prioritization concept are illustrated in Figure 1. Individual aptamer candidates are evaluated separately in a singleplex format using target-immobilized magnetic beads (Figure 1A). After secondary structure formation, each aptamer solution is repeatedly passed through a microspin filter unit containing the magnetic beads, thereby allowing aptamer–target binding to occur. Aptamers not retained on the target-immobilized beads are recovered in the flow-through fraction and quantified (Figure 1B).

After repeated sample passage, fresh assay buffer is sequentially applied to the microspin filter unit to promote aptamer dissociation from the target-immobilized beads. Because most of each wash volume is driven through the bead layer by centrifugation and collected, relatively little liquid remains in contact with the beads between cycles. This was the main reason for adopting a centrifugal filter rather than magnetic separation, in which a residual film and dead volume persist around the beads after the supernatant is removed and dissociated aptamer retained in that volume may rebind to the immobilized target [35]. During repeated washing, rapidly dissociating aptamers are expected to be preferentially removed, whereas slowly dissociating aptamers are expected to remain bound to the target-immobilized beads for a longer period (Figure 1C). After washing, aptamers retained on the beads are recovered by elution and quantified by qPCR.

Aptamer retention after washing is evaluated using W0-NRT. This metric represents the fraction of the bead-associated aptamer population estimated at the end of the repeated sample-passage step, based on the difference between the input and flow-through amounts, that is recovered in the final eluate after washing (Figure 1D). Wash 0 corresponds to the state immediately before the first wash cycle and does not represent the state immediately after initial binding, because both association and dissociation can occur during repeated sample passage. By normalizing to this end-of-passage reference, W0-NRT reduces the influence of differences in capture during sample passage and focuses on retention during washing. Although retention during washing can be influenced by multiple experimental factors, dissociation kinetics are expected to be a major determinant of differences in retention after accounting for differences in capture during sample passage. The expected inverse relationship between retention and *k*_off_ is schematically illustrated in Figure 1D and is experimentally evaluated in subsequent analyses using SPR-derived *k*_off_ values.

### 3.2. Establishment of MSF–k_off_ Assay Conditions Using a Model Aptamer Pair

**Figure 2:**
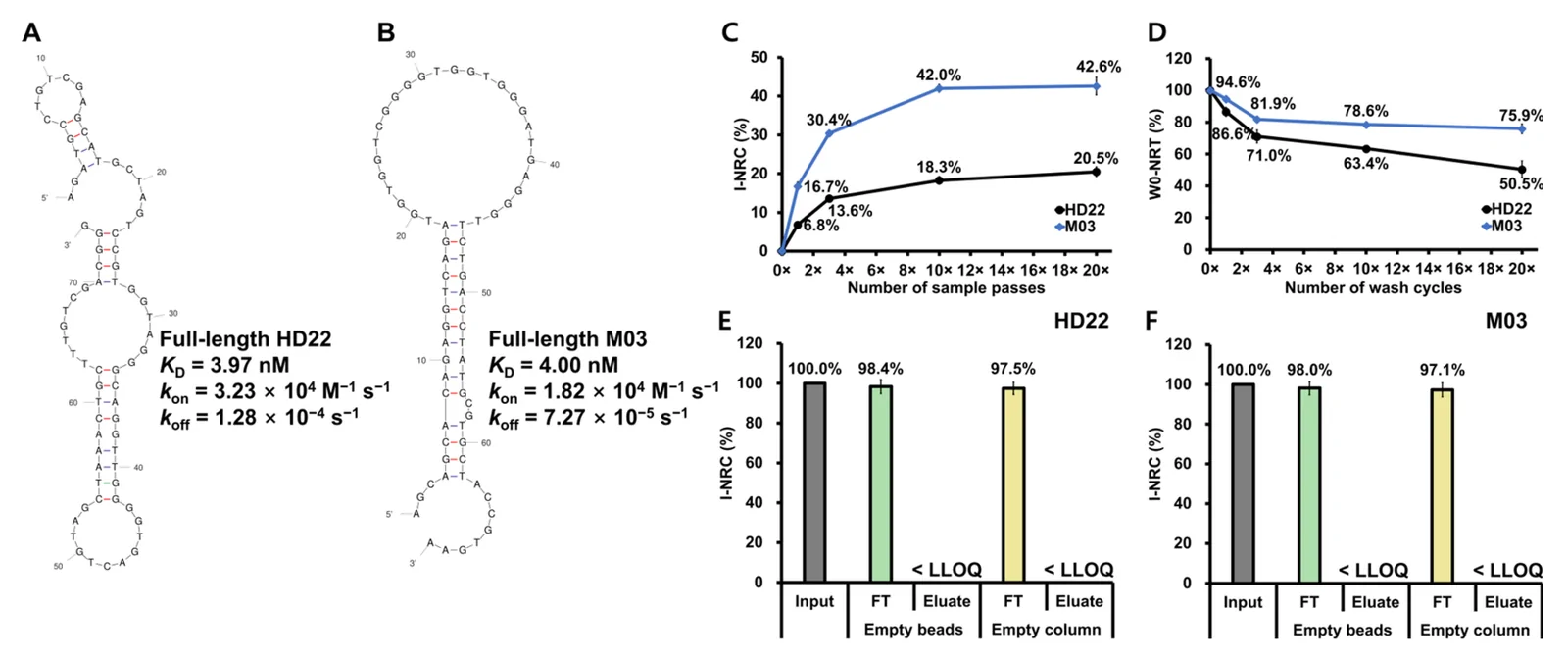
Establishment of MSF–*k*_off_ assay conditions for subsequent W0-NRT–*k*_off_ correlation analysis. (**A**,**B**) mfold-predicted secondary structures and SPR-derived affinity and kinetic parameters of full-length (**A**) HD22 and (**B**) M03. (**C**) I-NRC of HD22 and M03 after 0, 1, 3, 10, and 20 sample passages. The value at 0 sample passages was set to zero as a graphical reference and was not experimentally measured. (**D**) W0-NRT of HD22 and M03 after 0, 1, 3, 10, and 20 wash cycles. (**E**,**F**) I-NRC of HD22 (**E**) and M03 (**F**) under empty-bead and empty-microspin filter unit control conditions. Empty beads did not contain immobilized thrombin, whereas empty microspin filter unit contained neither beads nor thrombin. Data in (**C**–**F**) are presented as the mean ± SD of three parallel process replicates (*n* = 3). Some error bars are smaller than the data symbols. Values below the lower limit of quantification are indicated as <LLOQ. I-NRC indicates input-normalized recovery; W0-NRT, Wash 0-normalized retention; FT indicates flow-through, and LLOQ indicates the lower limit of quantification.

To establish the experimental conditions for the MSF–*k*_off_ assay, HD22 and M03 were selected as a model thrombin aptamer pair. The full-length sequences of the two aptamers exhibited different mfold-predicted secondary structures (Figure 2A,B). In SPR analysis, HD22 and M03 showed nearly identical *K*_D_ values of 3.97 and 4.00 nM, respectively, whereas their *k*_off_ values differed, with values of 1.28 × 10^−4^ and 7.27 × 10^−5^ s^−1^, respectively. Their *k*_on_ values also differed to some extent, with values of 3.23 × 10^4^ and 1.82 × 10^4^ M^−1^ s^−1^, respectively; however, because their *K*_D_ values were nearly identical, their overall binding affinities were maintained at a comparable level. Therefore, this aptamer pair was considered suitable for evaluating whether differences in dissociation kinetics are reflected in MSF retention under conditions that minimize the influence of affinity differences. Using this model pair, we evaluated the major experimental parameters of the MSF–*k*_off_ assay, including the number of sample passages and wash cycles.

For both aptamers, I-NRC increased as the number of sample passages increased (Figure 2C). The recovery of HD22 increased from 6.8% after one passage to 13.6%, 18.3%, and 20.5% after 3, 10, and 20 passages, respectively. The corresponding recovery values for M03 were 16.7%, 30.4%, 42.0%, and 42.6%, respectively. These I-NRC values do not directly represent the binding fraction expected at equilibrium. During sample passage, the aptamer solution is repeatedly passed through the bead layer by centrifugation rather than being incubated with the target for an extended period. Therefore, contact with immobilized thrombin during each passage is limited, and aptamer recovery accumulates over repeated passages. Accordingly, I-NRC reflects the fraction of aptamer recovered relative to the input under repeated short-contact conditions, rather than the equilibrium bound fraction predicted from *K*_D_. Although 20 passages resulted in the highest recovery for both aptamers, the additional increase after 10 passages was limited. In particular, for M03, recovery increased by only 0.6 percentage points when the number of passages was increased from 10 to 20. Therefore, 10 sample passages were considered to provide relatively high recovery among the tested conditions without excessively increasing the assay time. Accordingly, 10 sample passages were used in subsequent assays as a practical operational condition.

W0-NRT progressively decreased as the number of wash cycles increased (Figure 2D). After 1, 3, 10, and 20 wash cycles, HD22 showed W0-NRT values of 86.6%, 71.0%, 63.4%, and 50.5%, respectively. M03 consistently showed higher W0-NRT, with corresponding values of 94.6%, 81.9%, 78.6%, and 75.9%, which was consistent with its lower SPR-derived *k*_off_ value. The difference in W0-NRT between the two aptamers increased with repeated washing, reaching 15.2 percentage points after 10 wash cycles and 25.4 percentage points after 20 wash cycles. Although 20 wash cycles further increased the difference between the two aptamers, a clear W0-NRT difference consistent with the SPR-derived dissociation kinetics was already observed after 10 wash cycles. Because increasing the number of wash cycles from 10 to 20 doubled the sample-processing burden associated with the washing step, 10 wash cycles were selected as a practical assay condition that allowed discrimination of W0-NRT differences between candidates without further extending the experimental procedure.

To determine whether aptamer retention depended on immobilized thrombin, empty-bead and empty-microspin filter unit controls were evaluated (Figure 2E,F). For HD22, 98.4% and 97.5% of the input were recovered in the flow-through fractions of the empty-bead and empty-microspin filter unit controls, respectively. The corresponding values for M03 were 98.0% and 97.1%, respectively. Aptamer levels in all control eluates were below the lower limit of quantification. Thus, no quantifiable aptamer retention was observed in the absence of immobilized thrombin, and most of the input was recovered in the flow-through fraction. These results more clearly indicate that the retention measured in the assay depended on the presence of immobilized thrombin rather than on nonspecific retention by the magnetic beads or the microspin filter unit itself.

To further assess whether retention could arise from nucleotide composition or the constant regions of the aptamer construct, a scrambled HD22 control was evaluated in which the aptamer-derived region was randomly rearranged while preserving its nucleotide composition and the flanking constant regions (Figure S1). Under the standard assay conditions using thrombin-immobilized magnetic beads, 99.47% of the scrambled HD22 input was recovered in the flow-through fraction, whereas the signal in the final eluate was below the LLOQ. Together with the empty-bead and empty-microspin filter controls, these results indicate that the retention observed for HD22 was not attributable to nucleotide composition, the constant regions, or nonspecific retention by the beads or microspin filter unit.

The effects of the buffer matrix and oligonucleotide cleanup procedure on qPCR quantification were also evaluated (Figure S2). With the distilled-water condition without cleanup set to 100%, the relative recovery was 96.50% in 1× base reaction buffer without cleanup, 94.72% for distilled water after cleanup, and 94.27% for 8 M urea after cleanup. Recovery was therefore within 6% of the reference condition under all tested conditions, including the 8 M urea elution condition used in the MSF–*k*_off_ assay. The difference in relative recovery between the 1× base reaction buffer without cleanup and the 8 M urea condition after cleanup was 2.23 percentage points.

The Wash 0 amount used in the denominator of W0-NRT is estimated as the difference between the input and flow-through amounts, and this estimate was evaluated using a no-wash control. For AYA1809002, M09, and HD22, the estimated Wash 0 amounts were 91.14%, 67.76%, and 29.24% of the input, respectively, whereas the corresponding amounts measured by direct elution without washing were 90.81%, 65.66%, and 27.37% (Figure S3). The estimated values therefore exceeded the directly measured values by 0.33–2.10 percentage points of the input. This difference was similar in magnitude to the 1.6–2.9% of input that was not recovered in the flow-through in the empty-bead and empty-microspin filter unit controls, indicating that a small fraction of the input may be lost through nonspecific retention or other handling-related losses. Because this difference represents a larger relative contribution when the amount captured at Wash 0 is low, the use of the estimated Wash 0 amount may introduce greater relative error for candidates with lower capture. Nevertheless, the estimated and directly measured values showed the same ordering among the three aptamers.

Taken together, the W0-NRT differences observed under repeated washing conditions were consistent with the distinct dissociation behaviors of the two aptamers. However, a model pair consisting of only two aptamers was insufficient to establish a general relationship between W0-NRT and *k*_off_. Therefore, the relationship between W0-NRT and SPR-derived dissociation kinetics was further evaluated using a thrombin aptamer panel with diverse kinetic profiles.

### 3.3. Relationship Between W0-NRT and SPR-Derived Dissociation Rate Constants Across a Thrombin Aptamer Panel

Based on the results obtained using the model aptamer pair, we next evaluated the relationship between W0-NRT and dissociation kinetics in a panel of 13 thrombin aptamers with distinct kinetic profiles. The kinetic parameters of each aptamer were first characterized by SPR. Sensorgrams obtained over a concentration range of 5–500 nM were globally fitted to a 1:1 Langmuir binding model to determine *K*_D_, *k*_on_, and *k*_off_. The SPR-derived equilibrium dissociation constants and kinetic parameters are summarized in Table 1, and the corresponding sensorgrams and fitted curves for each aptamer are presented in Figure S4. Among these parameters, the SPR-derived *k*_off_ was used as a comparative kinetic estimate against which W0-NRT was evaluated.

These SPR measurements have several limitations that bear on how the derived parameters should be used. Each aptamer was measured as a single concentration series, so no replicate-based uncertainty is available, and the analysis software does not return standard errors for the globally fitted parameters. The 180 s dissociation phase is short relative to the interactions measured: the fitted *k*_off_ values correspond to half-lives of 27 to 411 min, and the fraction of the response predicted to be lost during the dissociation phase ranges from 0.50% to 7.46% (Table 1), so the slowest *k*_off_ values are the least well-constrained. The thrombin immobilization level of approximately 6000 RU is comparatively high, and mass-transport limitation, surface heterogeneity, and rebinding at the sensor surface cannot be excluded. Consistent with this, the 1:1 model does not describe every sensorgram equally well, and deviations between the measured and fitted curves are visible in Figure S4. In addition, the SPR running buffer and the base reaction buffer of the MSF–*k*_off_ assay have the same ionic composition, but the MSF assay buffer additionally contains 0.1% (*w*/*v*) BSA and 0.3 μM poly(dT)_30_, so the two assay environments are not fully matched. For these reasons, the SPR-derived values are treated here as comparative kinetic estimates obtained under the conditions of this study rather than as absolute kinetic constants.

To evaluate whether W0-NRT reflects differences in dissociation kinetics among thrombin aptamers, we first considered the theoretical relationship expected between aptamer retention after repeated washing and *k*_off_. During the washing process, fresh aptamer-free assay buffer passes through the bead layer, while aptamers dissociated from the target are continuously removed in the flow-through. Consequently, the concentration of free aptamer near the bead layer remains low, thereby limiting the likelihood that dissociated aptamers rebind to the immobilized target [35]. Therefore, under ideal conditions, if the decrease in aptamer–target complexes during washing is assumed to be governed predominantly by dissociation and to follow first-order dissociation kinetics [39], the retention rate R relative to Wash 0 can be expressed as follows:

(3) $$\mathrm{Retention}\;\mathrm{rate}\;R\;=\;e^{-k_{off}\;\times \;t_{eff}}$$

Here, R represents the ideal fraction of aptamer–target complexes retained relative to Wash 0, ranging from 0 to 1, and *k*_off_ denotes the dissociation rate constant. *t*_eff_ represents the effective dissociation time imposed by repeated washing. Rather than a single directly measured time interval, *t*_eff_ accounts for the cumulative exposure of the aptamer–target complexes to dissociation conditions as fresh wash buffer passes through the bead layer during each wash cycle. Taking the natural logarithm of the above equation gives:

(4) $$\ln(R)\;=\;{-k}_{\mathrm{off}}\;\times \;t_{\mathrm{eff}}$$

Equations (3) and (4) provide an idealized framework for the expected relationship between retention and *k*_off_ and were not fitted to the measured retention data as a mechanistic model.

**Figure 3:**
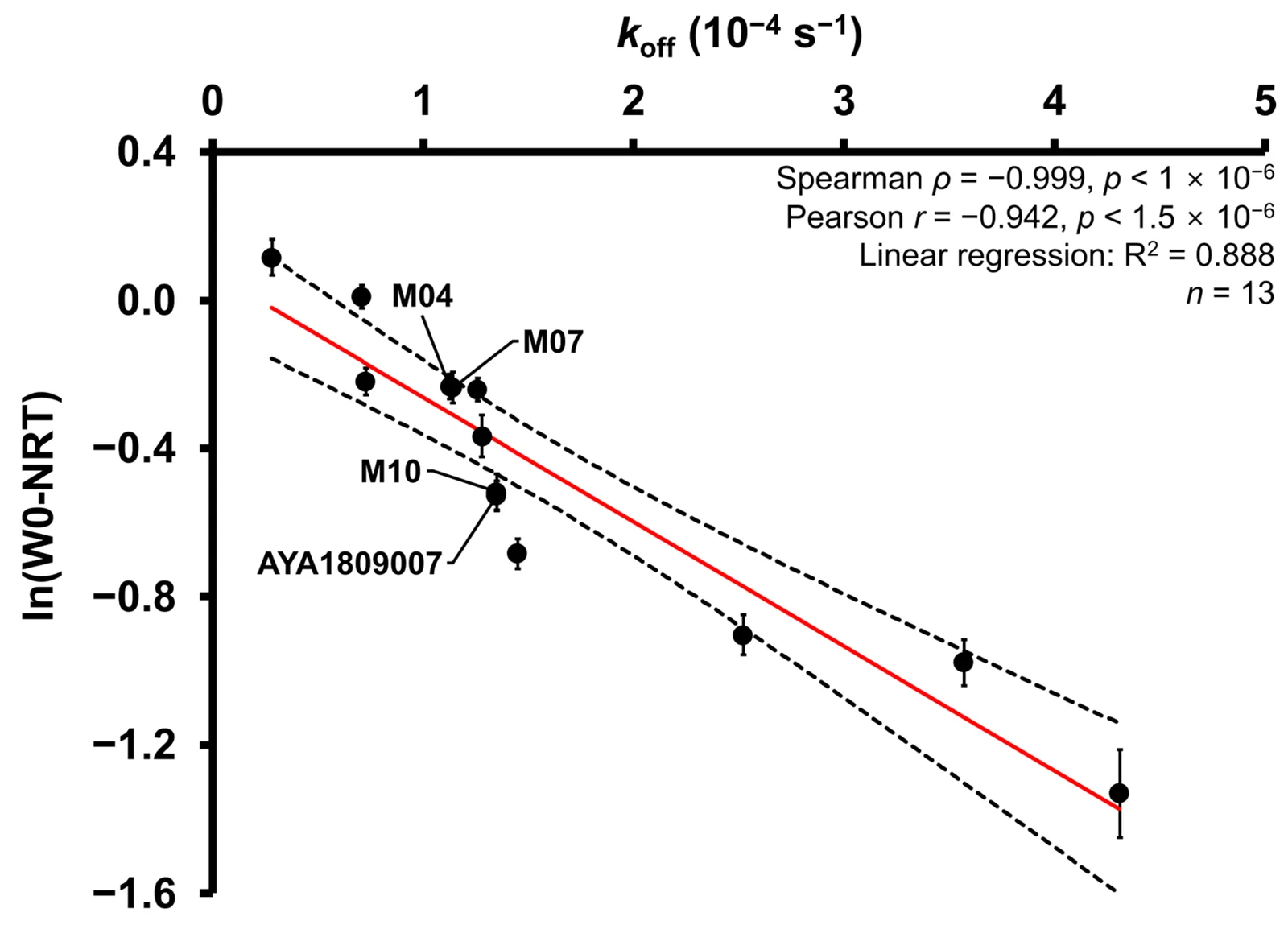
Relationship between ln-transformed W0-NRT and SPR-derived dissociation rate constants of thrombin aptamers. W0-NRT after 10 wash cycles was calculated for 13 thrombin aptamers, ln-transformed, and plotted against the SPR-derived dissociation rate constant (*k*_off_). Each point represents one aptamer. Two pairs of aptamers have nearly identical coordinates and therefore appear to overlap as a single symbol. The *k*_off_ values of M04 and M07 differ by 0.9%, whereas M10 and AYA1809007 have identical *k*_off_ values to three significant figures. These four aptamers are labeled in the plot, and the individual values for all 13 aptamers are provided in Table S2. W0-NRT values are shown as the ln-transformed mean retention obtained from three parallel process replicates, and the SD shown is that of the replicate W0-NRT values propagated through the logarithmic transformation. Each fraction was quantified by qPCR in technical triplicate. The red solid line represents the linear regression and the gray dashed lines represent the 95% confidence band of the fitted regression line. Spearman and Pearson correlation coefficients, the corresponding *p* values, *R*^2^, and the number of aptamers analyzed are indicated in the graph. Statistical significance of the Spearman correlation was assessed by a permutation test with 10^6^ resamples. Bootstrap 95% confidence intervals reported in the text were obtained by resampling the aptamers with 10,000 resamples.

Therefore, under identical target immobilization and washing conditions, aptamers with higher *k*_off_ values are expected to exhibit a greater decrease in retention after repeated washing, resulting in an inverse relationship between ln-transformed retention and *k*_off_. Because the experimentally determined W0-NRT may also be affected by the estimation of Wash 0, physical sample loss, and incomplete elution, it was treated as an operational estimate of R rather than as a direct measurement of the ideal retained fraction. W0-NRT was expressed as a fraction, ln-transformed, and plotted against the SPR-derived *k*_off_ values (Figure 3).

Consistent with the theoretical expectation, aptamers with lower SPR-derived *k*_off_ values exhibited higher W0-NRT values. The ln-transformed mean W0-NRT showed a nearly perfect inverse rank correlation with *k*_off_ (Spearman’s *ρ* = −0.999, permutation *p* < 1 × 10^−6^; bootstrap 95% CI, −1.000 to −0.979), as well as a strong inverse linear correlation (Pearson’s *r* = −0.942, *p* = 1.5 × 10^−6^, *R*^2^ = 0.888; bootstrap 95% CI, −0.983 to −0.855). In leave-one-out analysis, omitting any single aptamer left *ρ* between −0.998 and −1.000 and *r* between −0.905 and −0.962, indicating that the relationship was not driven by any single candidate. These results indicate that, within the thrombin panel, W0-NRT was closely associated with differences in dissociation behavior across aptamers with diverse kinetic profiles.

Because HD22 and M03 were used to establish the operating conditions of the assay before the panel was analyzed, the correlation was recalculated after excluding these two aptamers. Across the remaining eleven aptamers, the inverse relationship was essentially unchanged (Spearman’s *ρ* = −0.998, permutation *p* < 1 × 10^−6^; Pearson’s *r* = −0.941, *p* = 1.6 × 10^−5^, *R*^2^ = 0.885), and the empirical coefficient D was 3.39 × 10^3^ ± 0.41 × 10^3^ s, compared with 3.35 × 10^3^ ± 0.36 × 10^3^ s for the full panel. This analysis was performed as a sensitivity check rather than as an independent validation, because prospective evaluation using candidates characterized after the operating conditions were fixed was not performed in this study.

To assess whether the observed relationship depended on the least well-constrained SPR-derived values, the analysis was repeated after excluding the three aptamers for which the predicted decay during the 180 s dissociation phase was below 2% (M09, AYA1809003, and M03). Among the remaining ten aptamers, the relationship was essentially unchanged (Spearman’s *ρ* = −0.997, permutation *p* < 1 × 10^−6^; Pearson’s *r* = −0.934, *p* = 7.8 × 10^−5^, *R*^2^ = 0.872), indicating that the observed relationship did not depend on the *k*_off_ values that were least well-constrained by the SPR measurements.

Because *K*_D_, *k*_on_, and *k*_off_ are mathematically related through *K*_D_ = *k*_off_/*k*_on_, we next examined whether the relationship between W0-NRT and *k*_off_ might partly reflect equilibrium affinity. The ln-transformed W0-NRT correlated inversely with *K*_D_ (Pearson’s *r* = −0.865, *p* = 0.00014; Spearman’s *ρ* = −0.731, *p* = 0.0045) but showed no significant relationship with *k*_on_ (Pearson’s *r* = −0.409, *p* = 0.165; Spearman’s *ρ* = −0.473, *p* = 0.103). *k*_off_ and *K*_D_ were themselves correlated across the panel (Pearson’s *r* = 0.854, *p* = 0.00020; *r* = 0.833, *p* = 0.00041 after ln-transformation) (Table S3). In partial correlation analysis, the inverse relationship between *k*_off_ and ln-transformed W0-NRT was retained after adjustment for *K*_D_ (partial *r* = −0.780, *p* = 0.0028) and after adjustment for *k*_on_ (partial *r* = −0.932, *p* < 0.0001), whereas neither *K*_D_ nor *k*_on_ showed a significant relationship with ln-transformed W0-NRT after adjustment for *k*_off_ (partial *r* = −0.342, *p* = 0.276 and partial *r* = +0.166, *p* = 0.607, respectively). Multivariable regression gave a consistent result: in a model containing *k*_off_ and *K*_D_, only *k*_off_ contributed significantly (standardized *β* = −0.754, *p* = 0.0028 for *k*_off_ and *β* = −0.220, *p* = 0.277 for *K*_D_; *R*^2^ = 0.901; variance inflation factor = 3.70), and in a model containing *k*_off_ and *k*_on_, only *k*_off_ contributed significantly (*p* < 0.0001 for *k*_off_ and *p* = 0.607 for *k*_on_).

The panel also contains pairs of aptamers that are matched in one binding parameter while differing in others, allowing the same question to be examined at the level of individual candidates (Table S4). M10 and AYA1809007 have the same *k*_off_ to three significant figures (1.35 × 10^−4^ s^−1^) but differ approximately two-fold in both *K*_D_ (2.14 and 4.26 nM) and *k*_on_ (6.31 × 10^4^ and 3.17 × 10^4^ M^−1^ s^−1^); their W0-NRT values were 59.55 ± 2.94% and 59.00 ± 2.31% (*p* = 0.81). M04 and M07 differ by less than 1% in *k*_off_ while differing about 1.5-fold in *K*_D_ and *k*_on_, and gave W0-NRT values of 79.21 ± 2.61% and 79.01 ± 3.34% (*p* = 0.94). Conversely, HD22 and M03 have essentially identical *K*_D_ values (3.97 and 4.00 nM) but *k*_off_ values differing 1.76-fold and gave W0-NRT values of 69.33 ± 4.00% and 80.30 ± 3.01% (*p* = 0.022). M02 and M03, differing by 17% in *K*_D_ and 1.99-fold in *k*_off_, gave W0-NRT values of 50.47 ± 2.03% and 80.30 ± 3.01% (*p* = 0.0003). In these comparisons, W0-NRT followed *k*_off_ rather than *K*_D_ or *k*_on_. One pair behaved differently: HD22 and AYA1809001 differ by 2% in *k*_off_ but 2.2-fold in *K*_D_, and their W0-NRT values differed (69.33 ± 4.00% and 78.62 ± 2.38%, *p* = 0.036), indicating that factors other than *k*_off_ also contribute to the retention of individual candidates.

We also examined whether sequence-related properties contributed systematically to the observed retention. The 13 thrombin candidates comprised three construct families: HD22 (74 nt), six M-series aptamers (70–71 nt), and six AYA-series aptamers (85–86 nt), with GC contents ranging from 41.9% to 62.0%. The mean residuals from the ln(W0-NRT)–*k*_off_ regression were −0.011 for HD22, −0.030 for the M-series, and +0.032 for the AYA-series, with no significant difference between the two multi-member families (*p* = 0.49). The residuals were not significantly associated with sequence length (Pearson’s *r* = 0.24, *p* = 0.44) or GC content (*r* = −0.40, *p* = 0.18). Sequence length itself was correlated with *k*_off_ across the panel (*r* = 0.57, *p* = 0.043), reflecting the tendency of the longer AYA-series candidates to have higher *k*_off_ values; however, the absence of an association between sequence length and the regression residuals indicates that length did not account for the W0-NRT variation remaining after *k*_off_ was considered.

Differences in target-binding sites may also influence retention in the immobilized-target format. HD22 is reported to bind thrombin exosite II [40], whereas binding-site assignments are not available for all of the other aptamers evaluated here. Because thrombin is immobilized on the bead surface, the accessibility of a given binding site may depend on the orientation of the immobilized protein, and this effect may differ among aptamers directed toward different regions of the target. Differences in epitope accessibility could therefore contribute to W0-NRT in addition to intrinsic dissociation kinetics. The largest residual from the ln(W0-NRT)–*k*_off_ regression was 0.27 in ln units, corresponding to approximately a 24% difference in W0-NRT, indicating the scale of the unexplained variation within the present panel. W0-NRT-based ranking should therefore be interpreted cautiously when comparing aptamers that may recognize different binding sites on the same target.

These analyses do not establish that W0-NRT reflects *k*_off_ exclusively. The panel was assembled from previously characterized aptamers rather than designed so that *k*_off_ and *K*_D_ vary independently, the two parameters are correlated within it, and one of the matched pairs did not follow the general pattern. What the analyses do support is that, after accounting for *K*_D_ and *k*_on_ statistically and considering the individual matched comparisons, *k*_off_ remains associated with W0-NRT, whereas *K*_D_ and *k*_on_ do not show the same independent association in this panel. W0-NRT is therefore described here as a retention-based, dissociation-sensitive metric for comparing candidates under matched target and assay conditions rather than as a dissociation-specific readout, and prospective validation using candidate sets designed for independent variation in the binding parameters would be required to establish dissociation specificity directly.

The ideal expression in Equation (4) contains the product of *k*_off_ and *t*_eff_, such that the slope of ln(R) against *k*_off_ is −*t*_eff_, where *t*_eff_ represents the effective dissociation time. Because the present dataset was obtained under a single fixed washing condition, *t*_eff_ cannot be independently determined from the observed regression or separated from assay-dependent effects. In addition, W0-NRT may be affected by the estimation of Wash 0, physical sample loss, and incomplete elution, which can introduce assay-dependent scaling not included in the ideal first-order expression. We therefore described the observed relationship using the following explicitly empirical form:

(5) $$\mathrm{Retention}\;\mathrm{rate}\;R\;=\;A\;\times \;e^{-D\;\times \;k_{off}}$$

Taking the natural logarithm gives:

(6) $$\ln(R)\;=\;C-D\;\times k_{off}$$

Here, A is an empirical scaling factor, C is the corresponding intercept ln(A), and D is a positive empirical coefficient describing the relationship between ln-transformed retention and *k*_off_ under the fixed washing condition used in this study; the regression slope is therefore −D. Although D has units of time, it was not treated as an independently determined effective dissociation time because it also incorporates assay-dependent effects. For the thrombin panel, D was 3.35 × 10^3^ s (95% CI, 2.56 × 10^3^ to 4.15 × 10^3^ s), and C was 0.074 ± 0.071 and did not differ significantly from zero (*p* = 0.32). Weighted least-squares fitting, in which each aptamer was weighted by the inverse of the squared standard error of its ln-transformed W0-NRT, gave a similar estimate (D = 3.79 × 10^3^ ± 0.58 × 10^3^ s, compared with 3.35 × 10^3^ ± 0.36 × 10^3^ s by ordinary least squares), indicating that the estimate was not driven by differences in measurement uncertainty among aptamers.

To examine aptamer release during successive washes, the individual wash fractions W1–W10 were collected separately for M09, HD22, and AYA1809002, which span the *k*_off_ range of the thrombin panel, and quantified together with the flow-through and final eluate (Figure S5). Release during washing followed the order expected from *k*_off_. Relative to the estimated Wash 0 amount, the first wash released 0.7% of M09, 22.5% of HD22, and 41.3% of AYA1809002, while the cumulative release over ten washes was 1.0%, 32.3%, and 66.8%, respectively. Release fell below the LLOQ after four wash cycles for M09, seven for HD22, and nine for AYA1809002. Total recovery, calculated as the sum of the flow-through, individual wash fractions, and final eluate, ranged from 95.6% to 96.6% of the input. The unaccounted fraction therefore represented 3.4–4.4% of the input and showed no systematic relationship with *k*_off_.

The release profiles were not well described by a single exponential across all wash cycles. For AYA1809002, for example, the apparent per-cycle release constant decreased from 0.53 during the first wash to below 0.01 after the fifth wash, indicating that the early and late wash phases were not equivalent. The first one or two wash fractions may contain aptamer carried over in the residual liquid of the filter unit in addition to aptamer released by dissociation. We therefore did not fit the wash-fraction profiles to a single first-order kinetic model. To examine whether the candidate ordering depended on the early wash fractions, W0-NRT was recalculated by treating either the first wash or the first two washes as carryover and subtracting them from the estimated Wash 0 amount. The recalculated W0-NRT values were 93.4%, 73.8%, and 44.6% when W1 was excluded, and 93.5%, 79.4%, and 59.3% when W1 and W2 were excluded, for M09, HD22, and AYA1809002, respectively. The ordering remained M09 > HD22 > AYA1809002 in both analyses.

When the washing step was considered as a whole rather than cycle by cycle, the overall loss remained consistent with dissociation-dependent retention. The ln-transformed loss relative to Wash 0 was 0.010 for M09, 0.390 for HD22, and 1.103 for AYA1809002, and was proportional to *k*_off_ across the three aptamers (*R*^2^ = 0.988), corresponding to a proportionality constant of 2.6 × 10^3^ s. A separate estimate based on the wash-cycle titration of HD22 and M03 in Figure 2D gave 2.9 × 10^3^ s for ten wash cycles. These values are similar in magnitude to the empirical coefficient D obtained from the thrombin-panel regression (3.35 × 10^3^ s), supporting the interpretation that D reflects, at least in part, the cumulative exposure of the aptamer–target complexes to dissociation conditions during washing. Because these estimates were obtained from only a small number of aptamers and differed by up to approximately 30%, they were used only as a consistency check and not as an independent determination of an effective dissociation time.

The empirical relationship was therefore used to describe the association between W0-NRT and *k*_off_ under the fixed assay conditions rather than to derive absolute *k*_off_ values from W0-NRT. Within the thrombin panel, lower *k*_off_ values were associated with greater retention, supporting the use of W0-NRT for relative candidate ranking under matched target and assay conditions.

For two aptamers, the calculated W0-NRT slightly exceeded 100% (M09, 112.30%; AYA1809003, 101.01%; Figure 3). Because W0-NRT is calculated relative to the estimated Wash 0 amount, values above 100% are consistent with underestimation of this denominator and illustrate the uncertainty associated with the Wash 0 estimate. The values were retained as measured rather than truncated. Replacing the M09 value with either of the two independently measured values obtained in the day-to-day experiment (Table S5) changed the correlation with *k*_off_ only marginally, indicating that the overall relationship was not dependent on this value.

Day-to-day reproducibility was evaluated for M09, HD22, and AYA1809002 by repeating the complete workflow on two separate experimental days. The W0-NRT values on Days 1 and 2 were 92.73 ± 3.44% and 99.76 ± 3.01% for M09, 57.21 ± 3.20% and 65.32 ± 4.70% for HD22, and 26.48 ± 1.11% and 24.10 ± 2.01% for AYA1809002, respectively (Table S5). Within-day CVs ranged from 3.02% to 8.34% for these three aptamers, while the between-day CVs were 5.17%, 9.36%, and 6.66% for M09, HD22, and AYA1809002, respectively. Because aptamer folding, bead preparation, thrombin immobilization, and the qPCR standard curve were renewed together on each experimental day, between-day and between-batch variability could not be estimated separately. Inter-operator variability was not assessed because all experiments were performed by a single operator.

The candidate ranking was M09 > HD22 > AYA1809002 on both experimental days (Table S5), indicating that the relative ordering of these three candidates was reproduced across the two independent runs. However, because only three aptamers were included in the day-to-day comparison, this result should be interpreted as a limited assessment of ranking reproducibility.

When I-NRC was compared across different numbers of sample passages using the model aptamer pair, M03, which exhibited a lower SPR-derived *k*_off_ than HD22, showed higher I-NRC at all tested passage numbers—1, 3, 10, and 20 passages (Figure 2C). To determine whether *k*_off_ also contributed to I-NRC, we further examined the relationship between I-NRC and *k*_off_ across the panel of 13 thrombin aptamers. However, no significant correlation was observed for the untransformed data or after ln-transformation of I-NRC, *k*_off_, or both variables (Figure S6A–D). This finding should be interpreted in the context of the sample-passage step. Rather than being incubated with the target until equilibrium is reached, the aptamer solution repeatedly passes through the bead layer, resulting in limited contact time during each passage. Aptamer recovery during sample passage may therefore be influenced not only by association with the target but also by mass transport to the bead surface and dissociation of the formed aptamer–target complexes. Accordingly, I-NRC reflects the combined outcome of multiple kinetic processes occurring during repeated sample passage rather than a readout determined solely by *k*_off_. Across the panel of 13 thrombin aptamers, I-NRC showed no significant correlation with *K*_D_ (Spearman’s *ρ* = −0.017, *p* = 0.957; Pearson’s *r* = 0.154, *p* = 0.616), but showed a significant positive correlation with *k*_on_ (Spearman’s *ρ* = 0.846, *p* = 0.00027; Pearson’s *r* = 0.733, *p* = 0.0044) (Figure S6E,F). This association was retained in the leave-one-out analysis (*ρ* = 0.818–0.902, *p* ≤ 0.0011). In addition, partial correlation analysis showed that the association between I-NRC and *k*_on_ remained after adjustment for *k*_off_ (partial *r* = 0.657, *p* = 0.020), whereas no significant association was observed between I-NRC and *k*_off_ after adjustment for *k*_on_ (partial *r* = 0.166, *p* = 0.606).

These results suggest that, under the short sample-passage conditions used here, I-NRC may be influenced more strongly by association-related processes than by equilibrium affinity. Therefore, the relatively low I-NRC values observed for some high-affinity aptamers are not inconsistent with their *K*_D_ values. In contrast, W0-NRT is normalized to the aptamer population estimated to remain on the beads at the end of the sample-passage step, thereby reducing the influence of differences in capture during sample passage and allowing retention during washing to be compared. Thus, the difference in I-NRC observed for the model aptamer pair could not be explained solely by *k*_off_, and I-NRC was not considered a dissociation-focused readout.

### 3.4. Application of the MSF–k_off_ Assay for Post-SELEX Candidate Prioritization

To determine whether the relationship between W0-NRT and dissociation kinetics showed a consistent trend beyond the thrombin system, six streptavidin aptamers were evaluated using streptavidin-coated magnetic beads. Previously reported *k*_off_ values ranging from 3.60 × 10^−4^ to 5.10 × 10^−3^ s^−1^ were used as literature-based kinetic references [41]. These values were not measured in this study, and the constructs, buffer composition, temperature, immobilization orientation, and surface density used in the original report differed from those of the MSF–*k*_off_ assay. They were therefore used to provide a literature-reported kinetic ranking rather than as kinetic values measured under conditions matched to the present assay. After 10 wash cycles, the W0-NRT values ranged from 1.12% to 25.44% (Table S6). Direct comparison of the two regressions would require measurement of *k*_off_ for both target systems under matched conditions.

**Figure 4:**
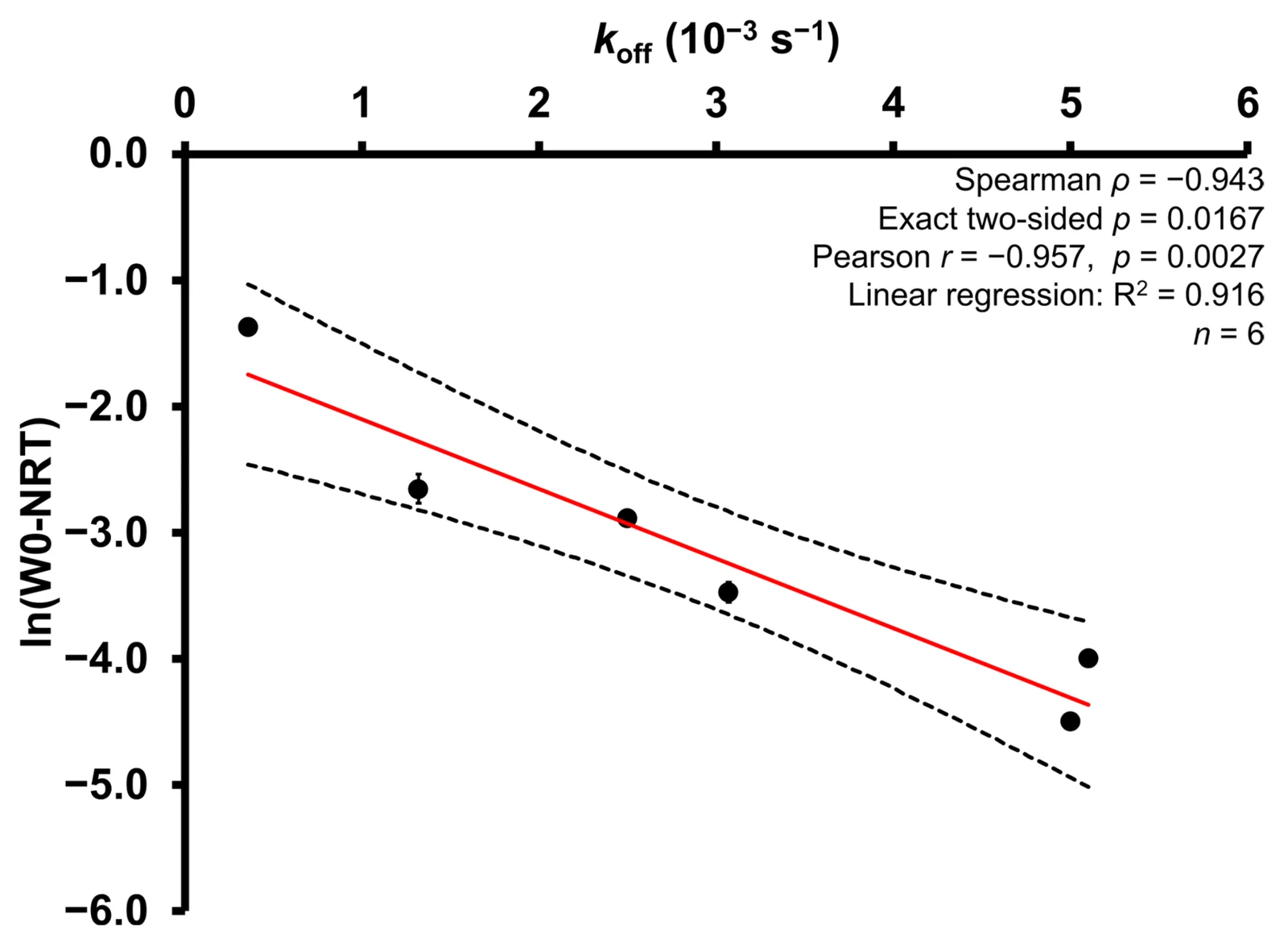
Relationship between ln-transformed W0-NRT and the literature-reported dissociation rate constants of streptavidin aptamers. W0-NRT after 10 wash cycles was calculated for 6 streptavidin aptamers, ln-transformed, and plotted against the dissociation rate constants (*k*_off_) reported in the literature. Each point represents one aptamer. W0-NRT values are shown as the ln-transformed mean retention obtained from three parallel process replicates, and the SD shown is that of the replicate W0-NRT values propagated through the logarithmic transformation. Each fraction was quantified by qPCR in technical triplicate. The red solid line represents the linear regression, and the gray dashed lines represent the 95% confidence band of the fitted regression line. Spearman and Pearson correlation coefficients, the corresponding *p* values, *R*^2^, and the number of aptamers analyzed are indicated in the graph. The statistical significance of the Spearman correlation was assessed using an exact two-sided test based on exhaustive enumeration of all permutations.

Aptamers with lower literature-reported *k*_off_ values tended to exhibit higher W0-NRT values in the MSF–*k*_off_ assay (Figure 4). To quantify this relationship, the mean W0-NRT value for each aptamer was ln-transformed and analyzed against the literature-reported *k*_off_ values. The ln-transformed mean W0-NRT showed a significant inverse rank correlation with *k*_off_ (Spearman’s *ρ* = −0.943, exact two-sided *p* = 0.0167), as well as a strong inverse linear correlation (Pearson’s *r* = −0.957, *p* = 0.0027; *R*^2^ = 0.916). The bootstrap 95% confidence interval was −0.999 to −0.901 for Pearson’s *r* and −1.000 to −0.515 for Spearman’s *ρ*, and in leave-one-out analysis, *ρ* ranged from −0.900 to −1.000 and *r* from −0.941 to −0.974. Given the small number of aptamers in this panel, the rank correlation remains relatively uncertain, and these results provide preliminary support for consistency between W0-NRT-based ranking and the literature-reported kinetic ranking rather than independent validation of the relationship.

Within the streptavidin panel, ln-transformed W0-NRT was associated with the literature-reported *k*_off_ (Pearson’s *r* = −0.957, *p* = 0.0027) but not with the reported *K*_D_ (*r* = −0.458, *p* = 0.36) or *k*_on_ (*r* = −0.628, *p* = 0.18). In addition, *k*_off_ and *K*_D_ were not strongly correlated within this panel (*r* = 0.555, *p* = 0.25), unlike in the thrombin panel. This pattern is consistent with W0-NRT being more closely associated with dissociation behavior than with *K*_D_ or *k*_on_ in the streptavidin panel, although the small number of aptamers limits the strength of this comparison.

Although an inverse relationship between ln-transformed W0-NRT and *k*_off_ was observed in both the thrombin and streptavidin aptamer panels, the fitted relationships differed between the two target systems. According to the empirical linear model presented in Equation (6), −D and C represent the slope and intercept, respectively; because C = ln(A), A is the corresponding scaling factor. For the thrombin panel, D was 3.35 × 10^3^ s and A was 1.08, whereas for the streptavidin panel, D was 5.51 × 10^2^ s and A was 0.21. Weighted least-squares fitting gave 6.24 × 10^2^ ± 0.51 × 10^2^ s for the streptavidin panel, compared with 5.51 × 10^2^ ± 0.83 × 10^2^ s by ordinary least squares, so the difference between the two target systems was unchanged. Analysis of covariance across all 19 aptamers showed significant effects of both the interaction between *k*_off_ and target system (*p* = 0.0001) and the target-system term (*p* < 0.0001), indicating that the two fitted relationships were statistically distinct. Thus, D is specific to the target format and assay conditions under which it is obtained and should not be interpreted as a universal calibration parameter. 

The *k*_off_ ranges of the two panels overlapped only between 3.6 × 10^−4^ and 4.3 × 10^−4^ s^−1^, with only one aptamer from each panel falling within this interval. A regression fitted to all 19 aptamers would yield Pearson’s *r* = −0.947 and *R*^2^ = 0.897; however, this apparent agreement reflects two clusters occupying largely non-overlapping *k*_off_ ranges rather than a common relationship. A pooled regression was therefore not used. The two candidates with comparable *k*_off_ values across the systems, AYA1809002 (4.31 × 10^−4^ s^−1^) and StrepApt5 (3.60 × 10^−4^ s^−1^), showed similar W0-NRT values of 26.4% and 25.4%, respectively, but the fitted relationships diverged outside this narrow overlapping range.

The difference between the fitted relationships may have arisen, at least in part, from differences in the target immobilization format between the two systems. In the streptavidin aptamer system, streptavidin-coated magnetic beads themselves served as the target-bearing surface, whereas in the thrombin aptamer system, biotinylated thrombin was additionally immobilized on streptavidin-coated magnetic beads to generate the target-bearing beads. Differences in immobilization format may affect target density, distance from the bead surface, binding-site accessibility, orientation heterogeneity, and the probability of rebinding during washing [35,42]. A higher local density of accessible target sites may increase the likelihood that a dissociated aptamer rebinds before leaving the bead layer, which could make retention less sensitive to *k*_off_. Differences in target orientation and binding-site accessibility may also affect the measured retention. These surface-dependent factors may therefore contribute to the different W0-NRT–*k*_off_ relationships observed for the two target systems, in addition to differences in intrinsic dissociation kinetics. Accordingly, W0-NRT should be interpreted within a given target and assay configuration rather than compared directly across different target systems.

The choice of washing format was also examined directly. Following identical binding incubation, HD22 and M03 were subjected to either centrifugal microspin filter-based washing or magnetic washing. W10/W0 retention was 69.32 ± 5.13% for HD22 and 86.68 ± 1.15% for M03 with the centrifugal microspin filter format, compared with 81.65 ± 2.54% for HD22 and 91.17 ± 3.32% for M03 with magnetic separation (Figure S7). The difference in W10/W0 retention between M03 and HD22 was 17.36 percentage points with the filter format and 9.52 percentage points with magnetic separation, while the relative ranking (M03 > HD22) was preserved in both formats. This result is consistent with less complete removal of the liquid phase during magnetic washing, in which dissociated aptamer remaining in the residual volume may rebind during subsequent cycles [35]. A column-free format could nonetheless be less costly and more readily automated, and only one magnetic washing configuration was tested here.

Several additional assay variables were not examined systematically in this study. Soluble target competition was not performed, and the amount of immobilized thrombin was not varied. During each wash cycle, fresh aptamer-free buffer is applied and dissociated aptamer is removed in the flow-through, which is expected to limit rebinding; however, a soluble competition experiment and a target-density series would be required to assess the contribution of rebinding and target density directly. The day-to-day reproducibility experiment used independently prepared beads and a separate thrombin immobilization on each experimental day, and the relative ranking of the three tested aptamers was preserved. Because bead preparation and experimental day were varied together, however, the contribution of bead-batch variability could not be separated from other sources of between-day variation. For the streptavidin system, competition with free biotin could help determine whether retention depends on recognition of the biotin-binding pocket, but this control was not performed in the present study.

Meanwhile, the parallel MSF–*k*_off_ format allows multiple candidates to be processed in the same batch during post-SELEX candidate prioritization. The SPR analysis performed in this study used a single concentration series per aptamer and was estimated to require approximately 3 h per candidate, most of which was unattended instrument time. In the MSF–*k*_off_ assay, each filter unit undergoes 22 centrifugation steps, comprising one conditioning step, 10 sample passages, 10 washes, and one elution, followed by six additional centrifugation steps during oligonucleotide cleanup. These steps are performed in batches, and a 24-position microcentrifuge accommodates eight candidates in triplicate. Processing one such batch was estimated to require approximately 3.5 h, corresponding to approximately 26 min per candidate, although most of this period involves operator handling. The throughput benefit therefore applies within the capacity of the rotor; beyond this capacity, both elapsed time and hands-on effort increase with the number of candidates.

After excluding oligonucleotide synthesis costs common to both methods, the estimated assay-related cost was $37.84 for one MSF–*k*_off_ process replicate and $260.00 for one SPR concentration series (Archived version available online: https://perma.cc/6L3E-TDDB, accessed on 31 August 2026) [43]. On a three-versus-three basis, the corresponding costs were $69.79 and $780.00, respectively (Table S7). Both MSF–*k*_off_ estimates include the aptamer-specific standard curve and no-template control, which are required once per candidate irrespective of the number of process replicates. One-time SPR setup and target-preparation charges are listed separately in Table S7 and are not included in these per-candidate estimates. Because the SPR value represents an outsourced full-service analysis from a single provider, whereas the MSF–*k*_off_ estimate includes only the reagents and consumables used in our laboratory and excludes labor, instrument acquisition and amortization, maintenance, and equipment costs, these values should be regarded as illustrative local estimates rather than an equivalent economic comparison.

### 3.5. Application of MSF–k_off_ Ranking to a Wash-Based Sandwich Detection Assay

**Figure 5:**
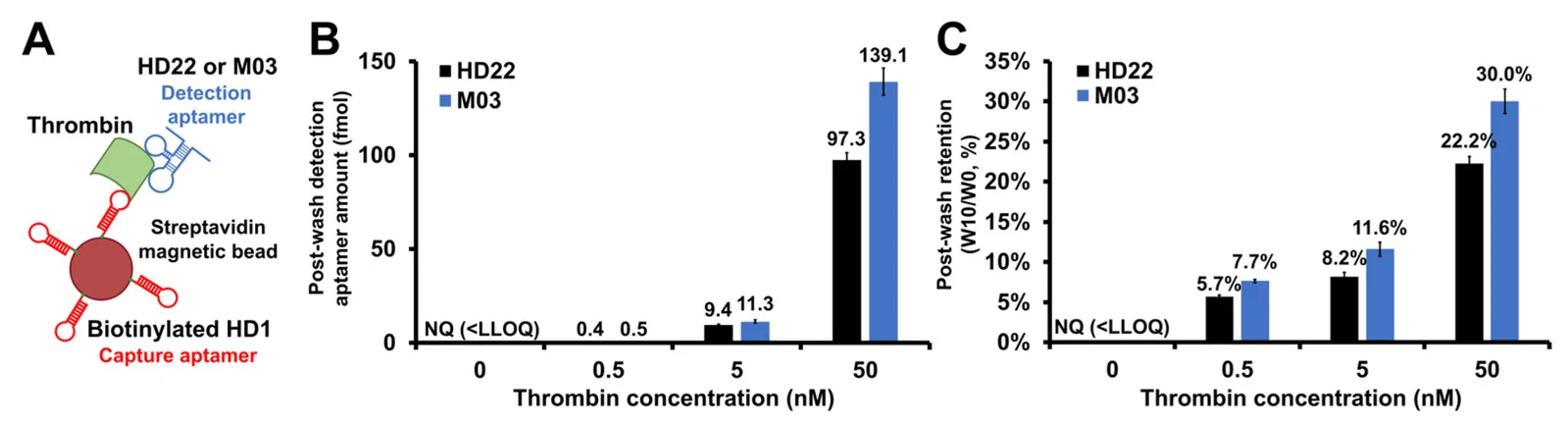
Sandwich detection of thrombin using aptamers ranked by the MSF–*k*_off_ assay. (**A**) Schematic of the sandwich assay configuration. Biotinylated HD1 was immobilized on streptavidin magnetic beads as the capture aptamer, and HD22 or M03 was used as the detection aptamer for thrombin. (**B**) Amount of detection aptamer recovered after ten washes at thrombin concentrations of 0, 0.5, 5, and 50 nM, as quantified by qPCR. (**C**) Post-wash retention of HD22 and M03, calculated as W10/W0 × 100 using the corresponding unwashed value at each thrombin concentration. Data are presented as the mean ± SD of three parallel assay replicates (*n* = 3), with each sample quantified by qPCR in technical triplicate. At 0 nM thrombin, the qPCR signals were below the lower limit of quantification for both detection aptamers and are indicated as NQ (<LLOQ). NQ, not quantifiable; LLOQ, lower limit of quantification.

To examine whether the relative ranking obtained with the MSF–*k*_off_ assay was preserved in a wash-based detection format, HD22 and M03 were compared as detection aptamers in a sandwich assay for thrombin (Figure 5A). The two aptamers have essentially identical SPR-derived equilibrium dissociation constants (3.97 and 4.00 nM, respectively) but differ 1.76-fold in *k*_off_, with M03 exhibiting the lower dissociation rate. Consistent with this difference, M03 was ranked above HD22 in the MSF–*k*_off_ assay, with W0-NRT values of 80.30 ± 3.01% and 69.33 ± 4.00%, respectively (*p* = 0.022). In the sandwich assay, biotinylated HD1 was immobilized on streptavidin magnetic beads as the capture aptamer, and HD22 or M03 was subsequently applied as the detection aptamer. HD1 and HD22 are known to bind distinct sites on thrombin, exosite I and exosite II, respectively [40]. Prior to the sandwich assay, the compatibility of HD1 and M03 for simultaneous thrombin binding was evaluated because the binding site of M03 has not been established. M03 binding after HD1 pre-incubation remained at 97.8% of that observed without HD1 pre-incubation, indicating that HD1 pre-binding did not substantially reduce M03 binding (Figure S8).

After ten washing steps, the amount of recovered detection aptamer increased with increasing thrombin concentration for both HD22 and M03 (Figure 5B). At 0.5, 5, and 50 nM thrombin, the recovered amounts of HD22 were 0.4, 9.4, and 97.3 fmol, respectively, whereas the corresponding amounts of M03 were 0.5, 11.3, and 139.1 fmol. At 0 nM thrombin, the qPCR signals for both detection aptamers were below the lower limit of quantification (LLOQ). These absolute amounts show a thrombin concentration-dependent response for each detection aptamer; comparison between HD22 and M03 was based on the within-aptamer W10/W0 normalization described below.

When post-wash retention was expressed as W10/W0, M03 consistently exhibited higher retention than HD22 at all quantifiable thrombin concentrations (Figure 5C). The W10/W0 values for HD22 and M03 were 5.7% and 7.7% at 0.5 nM, 8.2% and 11.6% at 5 nM, and 22.2% and 30.0% at 50 nM thrombin, respectively, corresponding to M03:HD22 retention ratios of 1.35, 1.41, and 1.35. The relative ordering of the two detection aptamers was therefore maintained across the concentration range and was consistent with the lower SPR-derived *k*_off_ and higher MSF–*k*_off_ W0-NRT of M03. In an independent control experiment performed at 50 nM thrombin, the complete sandwich condition produced quantifiable post-wash signals, with W10/W0 values of 24.8% for HD22 and 33.3% for M03 (Figure S9A), whereas omission of HD1, thrombin, or the detection aptamer resulted in signals below the LLOQ (Figure S9B).

This comparison was performed with a single target and a single affinity-matched aptamer pair in buffer, and the assay represents a laboratory-format proof-of-concept sandwich measurement rather than a fully developed sensor device. Analytical performance in serum, plasma, or other complex matrices was not evaluated. In addition, W10/W0 increased with thrombin concentration despite identical washing conditions, indicating that post-wash retention in this detection format is not determined by sensor-aptamer dissociation alone. Surface-density-dependent processes, including rebinding within the bead layer, may also contribute. Accordingly, the agreement between the MSF–*k*_off_ assay and the sandwich assay should be interpreted as preservation of candidate rank order rather than quantitative agreement in retention magnitude. Nevertheless, the consistently higher post-wash retention of M03 supports the practical relevance of retention-based kinetic pre-screening for prioritizing aptamer candidates before the development of sensing formats that include washing steps.

## 4. Conclusions

In this study, we developed the MSF–*k*_off_ assay, a retention-based, dissociation-sensitive pre-screening method that prioritizes aptamer candidates according to the amount retained after repeated centrifugal washing, quantified by qPCR. In a panel of 13 thrombin aptamers, ln-transformed W0-NRT showed a strong inverse relationship with SPR-derived *k*_off_. In addition, in a panel of six streptavidin aptamers, the W0-NRT-based ranking exhibited a trend consistent with the ranking based on the literature-reported *k*_off_ values. Because these *k*_off_ values were taken from the literature rather than measured under the conditions used here, and because the target was presented in a different format, this finding represents preliminary evidence of consistency with a reported kinetic ranking rather than validation of the assay in a second target system. The slopes and intercepts of the regression equations also differed significantly between the two systems, and W0-NRT therefore does not serve as a universal calibration metric for determining absolute *k*_off_ values; rather, it functions as a retention-based, dissociation-sensitive readout for comparing candidates under matched target and assay conditions.

Applied to a wash-based sandwich detection assay for thrombin, the two affinity-matched candidates were ranked in the same order by W0-NRT and by post-wash retention at every quantifiable target concentration. However, the absolute retention and the magnitude of the separation were not quantitatively predicted by *k*_off_, indicating that the correspondence was in rank order rather than in retention magnitude. The MSF–*k*_off_ assay is therefore intended as a step preceding sensor development, in which candidates are prioritized before detailed kinetic characterization and before a detection format is built around them.

The parallel microspin filter format enables the simultaneous evaluation of multiple candidates before detailed SPR or BLI analysis and was estimated to offer relative advantages in terms of both analysis time and assay-related cost. The results of this study should be interpreted as preliminary evidence of feasibility. In this study, 13 thrombin aptamers and 6 streptavidin aptamers were evaluated in a single laboratory, with *k*_off_ values spanning approximately a 15-fold range within each panel. In addition, the candidates were not selected prospectively but were assembled from previously characterized sequences. Future prospective validation using larger, blinded post-SELEX candidate sets, kinetic reference values measured by SPR or BLI under conditions matched to the MSF–*k*_off_ assay, and diverse targets and immobilization formats will be necessary to establish the generalizability of the assay. Such validation should use the same aptamer constructs, buffer composition, and temperature for the kinetic reference measurements and the MSF–*k*_off_ assay. Comparison of the same target immobilized using different chemistries would also help distinguish effects arising from target presentation from those intrinsic to the target itself. In addition, because the dissociation sensitivity of the assay may depend, at least in part, on the completeness of liquid exchange during washing, adapting the workflow to a column-free format that preserves effective liquid exchange could reduce cost and waste and improve compatibility with automated screening. Taken together, the MSF–*k*_off_ assay provides a practical kinetic pre-screening approach that bridges NGS-based candidate discovery and quantitative kinetic characterization, enabling the efficient prioritization of slow-dissociating candidates within the same target system without replacing SPR or BLI.

## Figures and Tables

**Table 1:** SPR-derived equilibrium dissociation constants, kinetic parameters, and fit-quality metrics of thrombin aptamers. For each aptamer, one concentration series ranging from 5 to 500 nM was measured and globally fitted using a 1:1 Langmuir binding model. χ^2^ and *R*_max_ are the values returned by the analysis software. The predicted decay represents the fraction of the fitted response expected to be lost during the 180 s dissociation phase and was calculated as [1 − exp(−*k*_off_ × 180)] × 100.

Aptamer	*K*_D_ (nM)	*k*_on_ (M^−1^ s^−1^)	*k*_off_ (s^−1^)	*R*_max_ (RU)	χ^2^ (RU^2^)	Predicted Decay During 180 s (%)	Sequence Reference
HD22	3.97	3.23 × 10^4^	1.28 × 10^−4^	423.13	195.83	2.28	[36]
M02	3.43	4.21 × 10^4^	1.45 × 10^−4^	614.40	929.18	2.58	[37]
M03	4.00	1.82 × 10^4^	7.27 × 10^−5^	601.82	84.97	1.30	
M04	2.65	4.25 × 10^4^	1.13 × 10^−4^	550.28	554.06	2.01	
M07	1.83	6.24 × 10^4^	1.14 × 10^−4^	353.82	139.43	2.03	
M09	0.74	3.78 × 10^4^	2.81 × 10^−5^	437.70	169.57	0.50	
M10	2.14	6.31 × 10^4^	1.35 × 10^−4^	497.78	307.29	2.40	
AYA1809001	1.79	7.01 × 10^4^	1.26 × 10^−4^	348.91	167.29	2.24	[38]
AYA1809002	6.41	6.72 × 10^4^	4.31 × 10^−4^	594.16	1945.31	7.46	
AYA1809003	2.41	2.95 × 10^4^	7.09 × 10^−5^	415.02	607.24	1.27	
AYA1809004	5.62	4.49 × 10^4^	2.52 × 10^−4^	582.23	675.45	4.43	
AYA1809005	6.76	5.28 × 10^4^	3.57 × 10^−4^	602.08	1158.45	6.22	
AYA1809007	4.26	3.17 × 10^4^	1.35 × 10^−4^	532.93	732.11	2.40	

χ^2^ is an absolute fit statistic in RU^2^ and should not be interpreted by itself as a measure of how well *k*_off_ is constrained.

## Data Availability

The data supporting the findings of this study are available from the corresponding author upon reasonable request.

## Supplementary Materials

The following supporting information can be downloaded at: https://www.mdpi.com/article/10.3390/bios16090510/s1, Figure S1: Evaluation of nonspecific retention using scrambled HD22, empty-microspin filter, and empty-bead controls; Figure S2: Evaluation of buffer matrix effects and oligonucleotide cleanup recovery in qPCR quantification; Figure S3: Comparison of the estimated retained amount and directly measured I-NRC under the no-wash (Wash 0) condition; Figure S4: SPR sensorgrams and global kinetic fitting of thrombin aptamers; Figure S5: Mass balance and aptamer release profiles across sequential wash cycles in the MSF–*k*_off_ assay; Figure S6: Relationship between SPR-derived kinetic parameters and input-normalized recovery; Figure S7: Comparison of centrifugal microspin filter and column-free magnetic separation washing formats; Figure S8: Effect of HD1 pre-binding on M03 binding to thrombin; Figure S9: Control experiments for the HD1-based thrombin sandwich detection assay; Table S1: Oligonucleotide sequences used in this study [44,45]; Table S2: Summary of MSF–*k*_off_ assay metrics and SPR-derived kinetic parameters for thrombin aptamers; Table S3: Correlation, partial correlation, and multivariable regression analyses of W0-NRT and SPR-derived equilibrium and kinetic parameters in the thrombin aptamer panel; Table S4: Comparison of thrombin aptamer pairs matched in one binding parameter while differing in other parameters; Table S5: Day-to-day reproducibility of W0-NRT and candidate ranking; Table S6: Summary of MSF–*k*_off_ assay metrics and the literature-reported kinetic parameters for streptavidin aptamers; Table S7: Comparison of estimated assay-related costs for SPR and the MSF–*k*_off_ assay.
