# Supplementary material for: Retention-Based, Dissociation-Sensitive Pre-Screening of Aptamers Using a Centrifugal Microspin Filter
Source: Biosensors (Basel). 2026 Sep 10;16(9):510. doi: 10.3390/bios16090510 (PMC13604723; doi:10.3390/bios16090510)
Supplement: Supplementary file 1 [file biosensors-16-00510-s001.zip › biosensors-4483818-supplementary.pdf]

biosensors

MDPI

Article

Retention-Based, Dissociation-Sensitive Pre-Screening of Aptamers Using a Centrifugal Microspin Filter

Cheeyoon Ahn , Hwayeon Jeong, Kyungjin Jeon and Cheulhee Jung *

Department of Biotechnology, College of Life Sciences and Biotechnology, Korea University, Seoul 02841, Republic of Korea; k59123@korea.ac.kr(C.A.); cca1987@korea.ac.kr(H.J.); 2021140203@korea.ac.kr(K.J.)
* Correspondence: damo363@korea.ac.kr; Tel.: +82-2-3290-3023

This file includes:

1. Figure S1. Evaluation of nonspecific retention using scrambled HD22, empty-microspin filter, and empty-bead controls.
2. Figure S2. Evaluation of buffer matrix effects and oligonucleotide cleanup recovery in qPCR quantification.
3. Figure S3. Comparison of the estimated retained amount and directly measured I-NRC under the no-wash (Wash 0) condition.
4. Figure S4. SPR sensorgrams and global kinetic fitting of thrombin aptamers.
5. Figure S5. Mass balance and aptamer release profiles across sequential wash cycles in the MSF–k_{off} assay.
6. Figure S6. Relationship between SPR-derived kinetic parameters and input-normalized recovery.
7. Figure S7. Comparison of centrifugal microspin filter and column-free magnetic separation washing formats.
8. Figure S8. Effect of HD1 pre-binding on M03 binding to thrombin.
9. Figure S9. Control experiments for the HD1-based thrombin sandwich detection assay.
10. Table S1. Oligonucleotide sequences used in this study.
11. Table S2. Summary of MSF–k_{off} assay metrics and SPR-derived kinetic parameters for thrombin aptamers.
12. Table S3. Correlation, partial correlation, and multivariable regression analyses of W0-NRT and SPR-derived equilibrium and kinetic parameters in the thrombin aptamer panel.
13. Table S4. Comparison of thrombin aptamer pairs matched in one binding parameter while differing in other parameters.
14. Table S5. Day-to-day reproducibility of W0-NRT and candidate ranking.
15. Table S6. Summary of MSF–k_{off} assay metrics and literature-reported kinetic parameters for streptavidin aptamers.
16. Table S7. Comparison of estimated assay-related costs for SPR and the MSF–k_{off} assay.

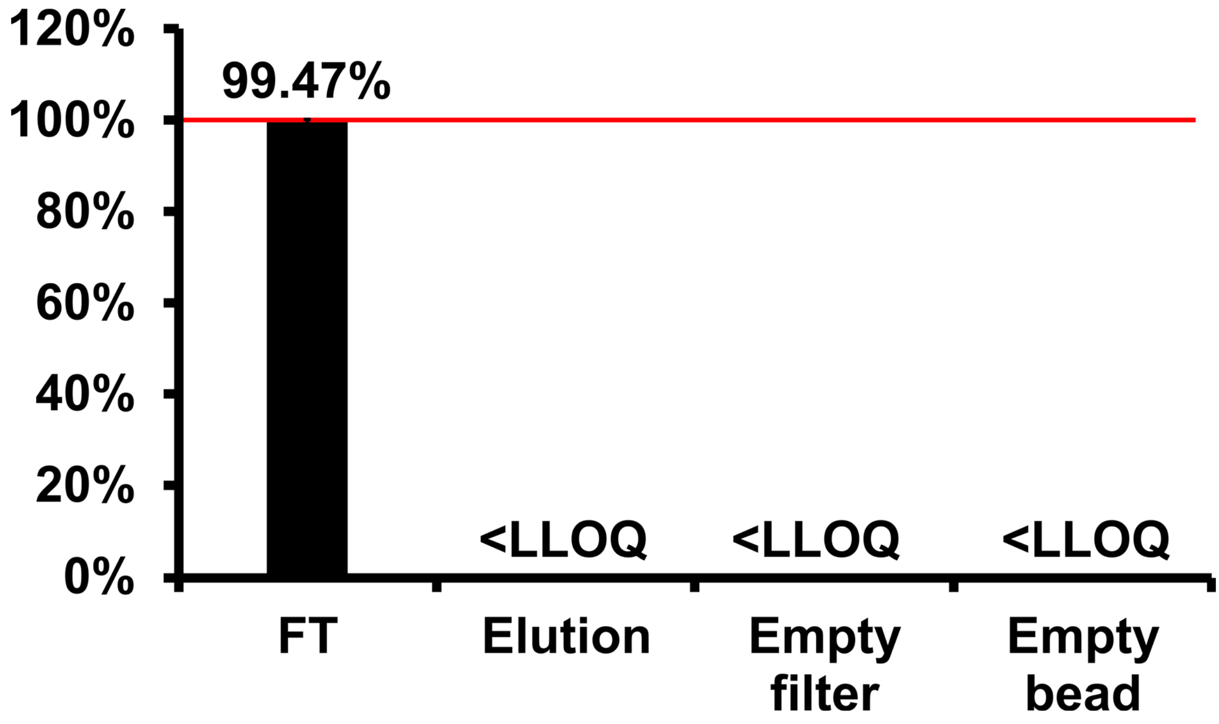

Figure S1. Evaluation of nonspecific retention using scrambled HD22, empty-microspin filter, and empty-bead controls. Nonspecific retention was evaluated using a scrambled HD22 sequence in which the nucleotide order was randomly rearranged while preserving the nucleotide composition of HD22 and the flanking constant regions. Under the standard assay conditions using thrombin-immobilized magnetic beads, 99.47% of the scrambled HD22 input was recovered in the flow-through (FT) fraction, whereas the signal in the final elution fraction was below the lower limit of quantification (LLOQ) of qPCR. The empty-microspin filter control consisted of a microspin filter unit without magnetic beads, whereas the empty-bead control used streptavidin magnetic beads without immobilized thrombin in the microspin filter unit. The retention-associated signals recovered from both controls were also below the LLOQ. Recovery in each condition was normalized to the corresponding input amount, which was set to 100%, and the red line indicates the 100% reference corresponding to the input. Data are presented as the mean ± SD of three parallel process replicates (n = 3).

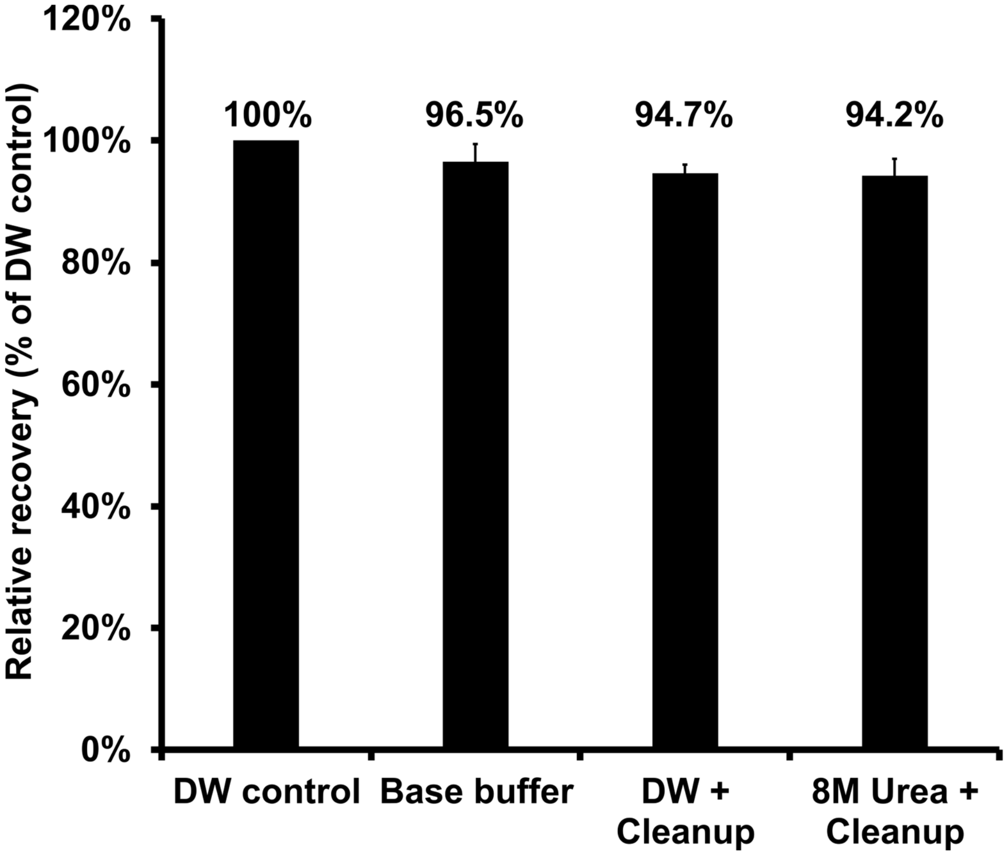

Figure S2. Evaluation of buffer matrix effects and oligonucleotide cleanup recovery in qPCR quantification. An amount of oligonucleotide equivalent to the input amount used in the MSF–k_{off} assay was prepared in distilled water (DW) or 1× base reaction buffer and quantified by qPCR. To evaluate recovery through the oligonucleotide cleanup procedure, the same amount of oligonucleotide was prepared in DW or 8 M urea, subjected to oligonucleotide cleanup, and subsequently quantified by qPCR. Recovery was normalized to the oligonucleotide prepared in DW without cleanup, which was set to 100%. The 8 M urea condition represents the elution condition used in the MSF–k_{off} assay. Data are presented as the mean ± SD (n = 3).

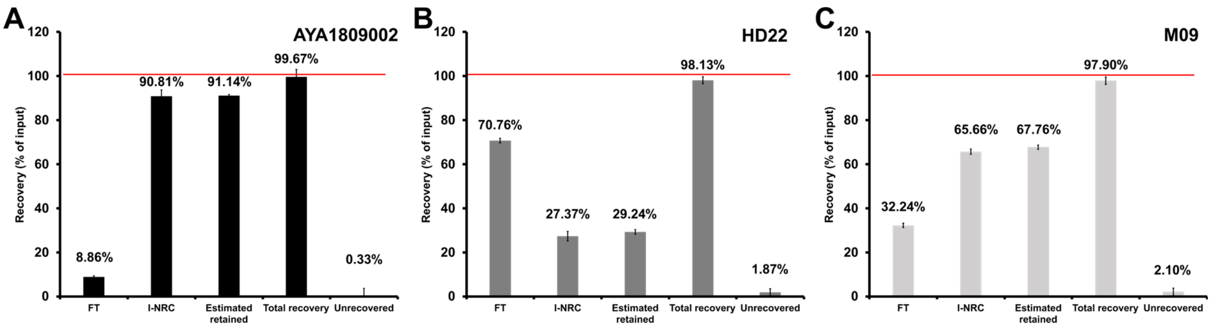

Figure S3. Comparison of the estimated retained amount and directly measured I-NRC under the no-wash (Wash 0) condition. Aptamer recovery under the no-wash (Wash 0) condition, in which aptamers were eluted directly without washing, was evaluated for AYA1809002 (**A**), HD22 (**B**), and M09 (**C**). Each fraction was normalized by setting the input amount for the corresponding experiment to 100%. FT represents the amount of aptamer recovered in the flow-through, and the estimated retained amount was calculated as 100 − FT (%). I-NRC represents the amount of aptamer recovered in the final elution fraction without washing, normalized to the input. Total recovery was calculated as the sum of FT and I-NRC, and the unrecovered fraction was calculated as 100 − total recovery. The red line indicates the 100% reference corresponding to the input. Data are presented as the mean ± SD of three parallel process replicates performed under the same experimental conditions (n = 3).

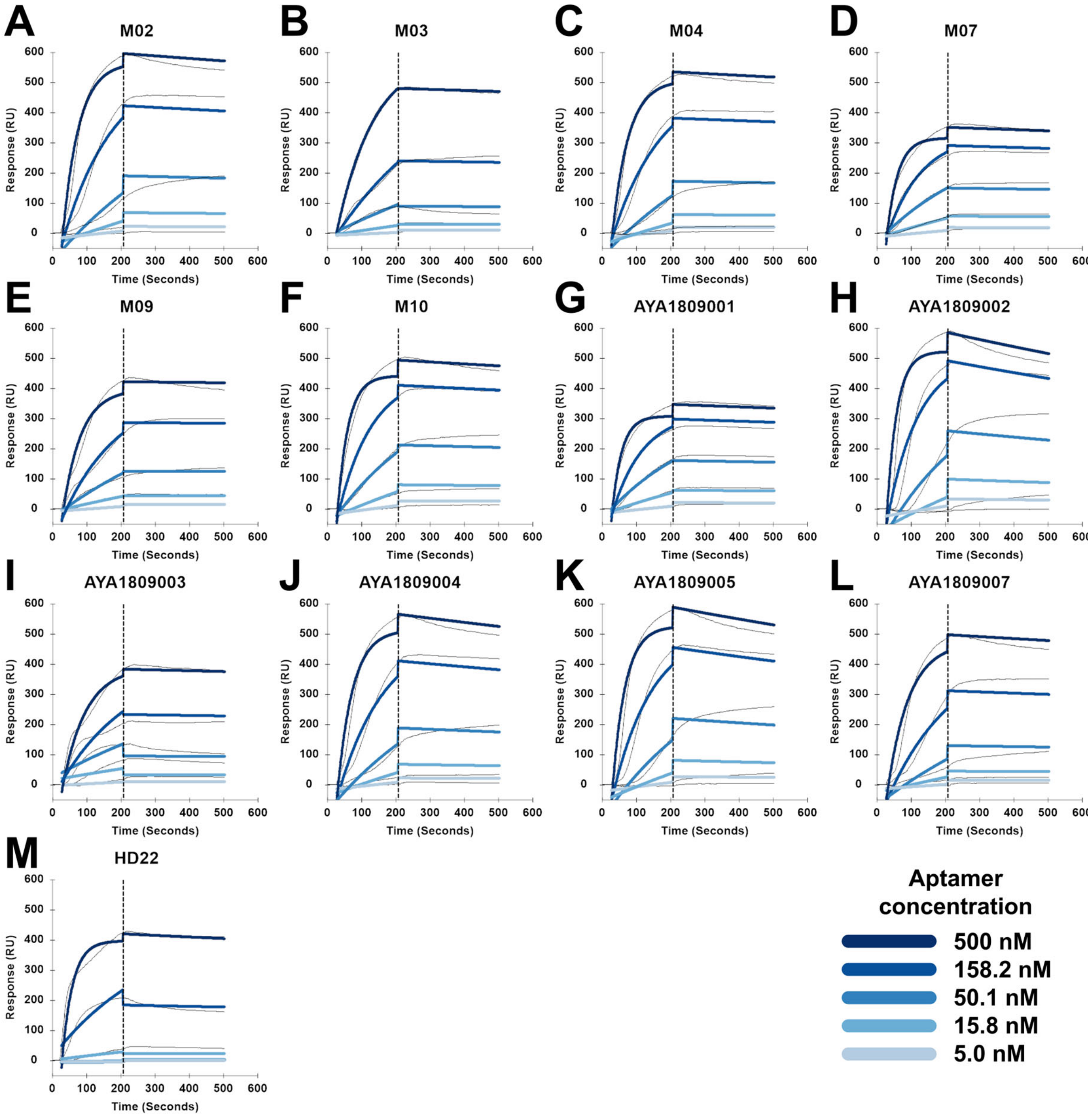

Figure S4. SPR sensorgrams and global kinetic fitting of thrombin aptamers. Reference- and blank-subtracted experimental SPR sensorgrams and globally fitted curves are shown for (**A**) M02, (**B**) M03, (**C**) M04, (**D**) M07, (**E**) M09, (**F**) M10, (**G**) AYA1809001, (**H**) AYA1809002, (**I**) AYA1809003, (**J**) AYA1809004, (**K**) AYA1809005, (**L**) AYA1809007, and (**M**) HD22. Each panel shows SPR sensorgrams for one of the 13 thrombin aptamers. Thin gray lines indicate experimentally measured sensorgrams, and colored lines indicate globally fitted curves. Vertical dashed lines indicate the transition from the association phase to the dissociation phase. Aptamers were injected over immobilized biotinylated native human α-thrombin at concentrations of 5.0, 15.8, 50.1, 158.2, and 500 nM. Each injection consisted of a 180 s association phase and a 180 s dissociation phase at a flow rate of 30 μL min^{-1}. Kinetic parameters were determined using a 1:1 Langmuir binding model.

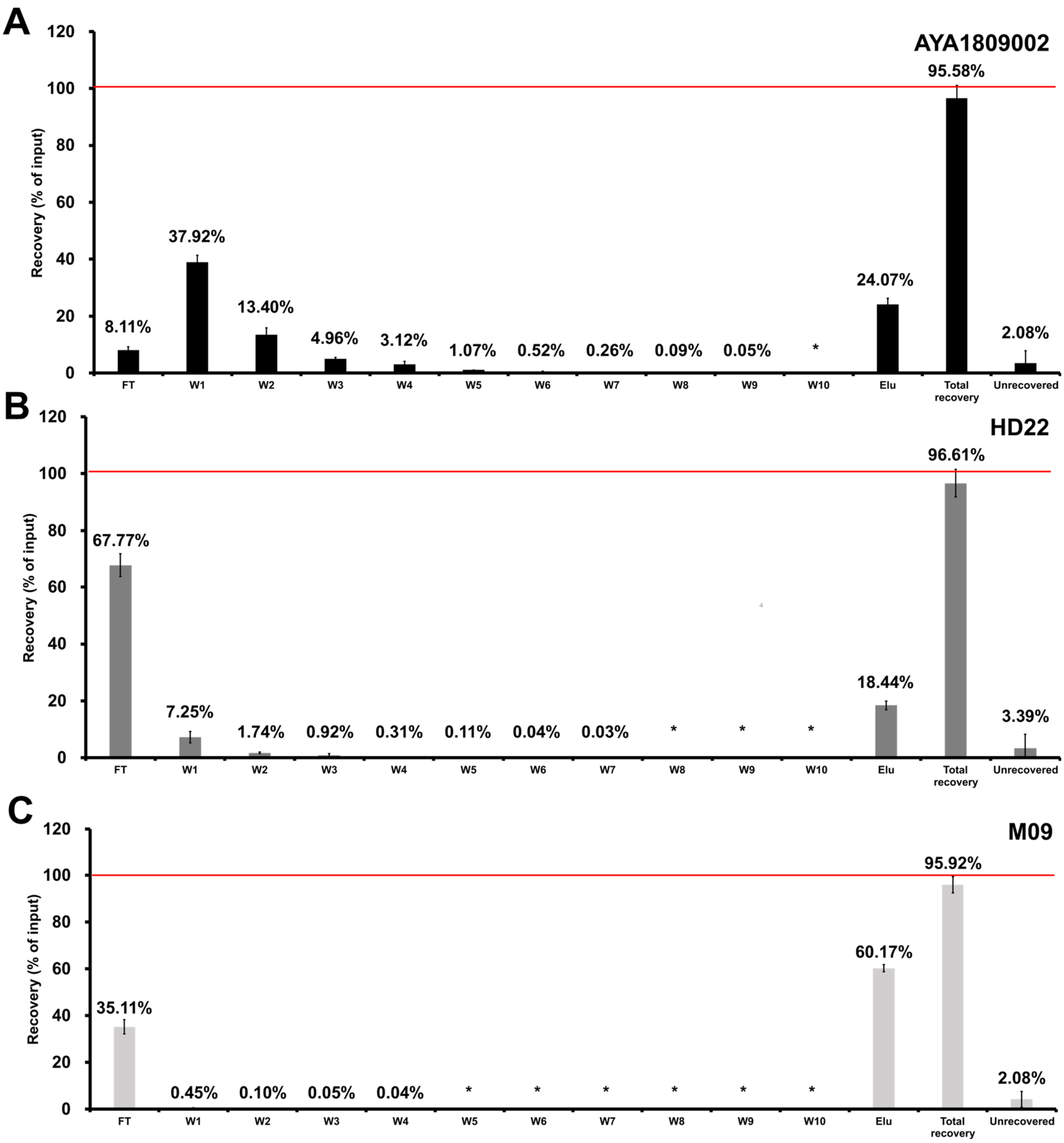

Figure S5. Mass balance and aptamer release profiles across sequential wash cycles in the MSF–k_{off} assay. Aptamer recovery was evaluated for AYA1809002 (**A**), HD22 (**B**), and M09 (**C**). The flow-through (FT), individual wash fractions (W1–W10), and final elution fraction were collected separately and quantified by qPCR. Each fraction was normalized to the corresponding input amount, which was set to 100%. Total recovery was calculated as the sum of the FT, W1–W10, and final elution fractions, and the unrecovered material was calculated as 100 − total recovery. Data are presented as the mean ± SD of three parallel process replicates (n = 3). The red line indicates the 100% reference corresponding to the input. Asterisks (*) indicate detected values below the lower limit of quantification.

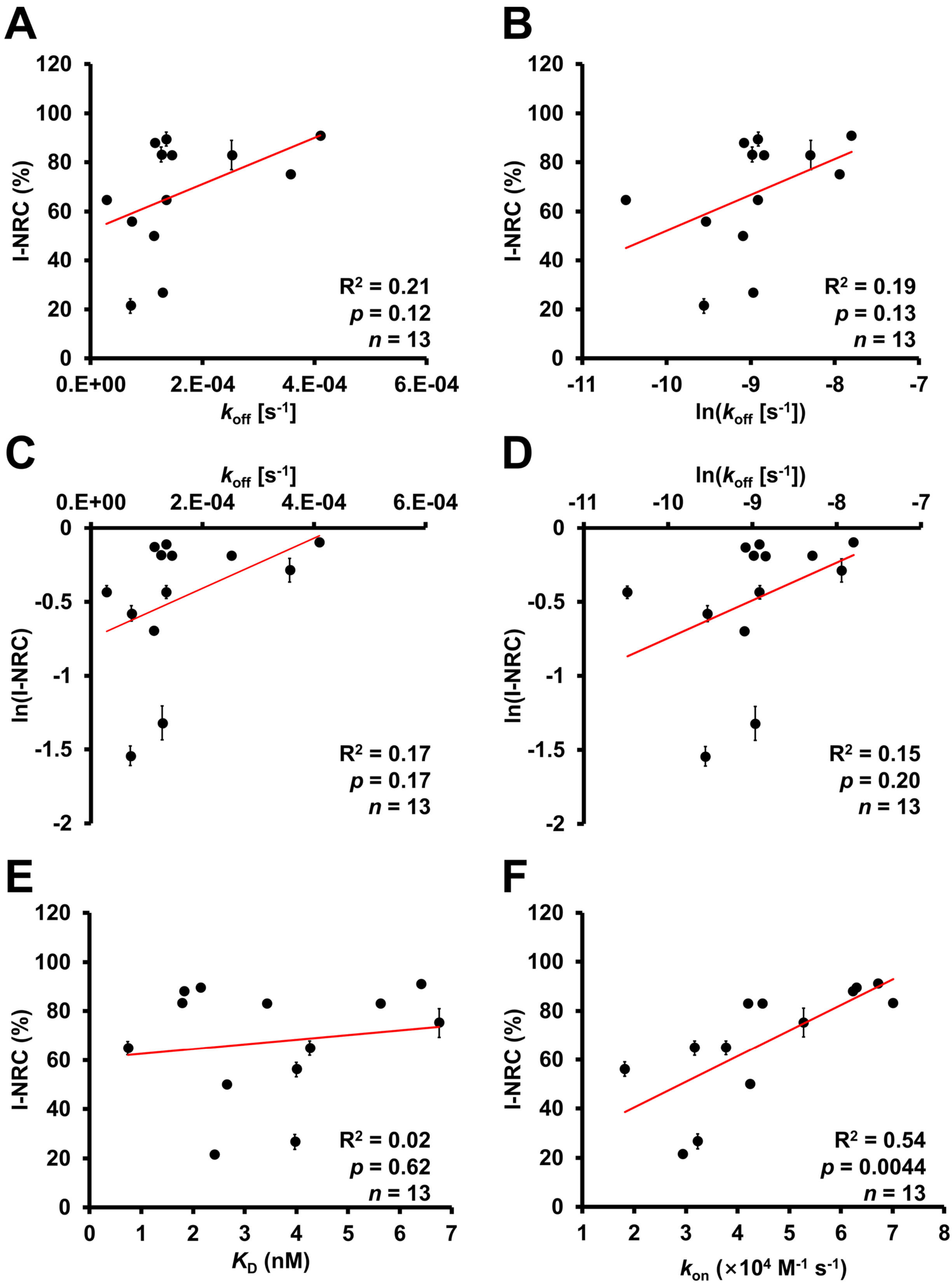

Figure S6. Relationship between SPR-derived kinetic parameters and input-normalized recovery. The relationship between SPR-derived kinetic parameters and input-normalized recovery (I-NRC), obtained from the MSF–k_{off} assay, was evaluated for 13 thrombin aptamers under different data transformation conditions. (**A**) The relationship between k_{off} and I-NRC. (**B**) The relationship between natural log-transformed k_{off} and I-NRC. (**C**) The relationship between k_{off} and natural log-transformed I-NRC. (**D**) The relationship between natural log-transformed k_{off} and natural log-

transformed I-NRC. (**E**) The relationship between K_D and I-NRC. (**F**) The relationship between k_{on} and I-NRC. For logarithmic transformation, I-NRC percentages were first converted to fractional values ranging from 0 to 1. Each point represents the mean value for an individual aptamer, and error bars indicate the standard deviation. Red lines represent unweighted simple linear regression fits. The coefficient of determination, two-sided p-value for the regression slope, and number of aptamers analyzed are indicated in each panel. No statistically significant linear relationship between k_{off} and I-NRC was observed under any of the tested transformation conditions (**A**–**D**). No significant linear relationship was observed between K_D and I-NRC (**E**), whereas a significant positive linear relationship was observed between k_{on} and I-NRC (**F**).

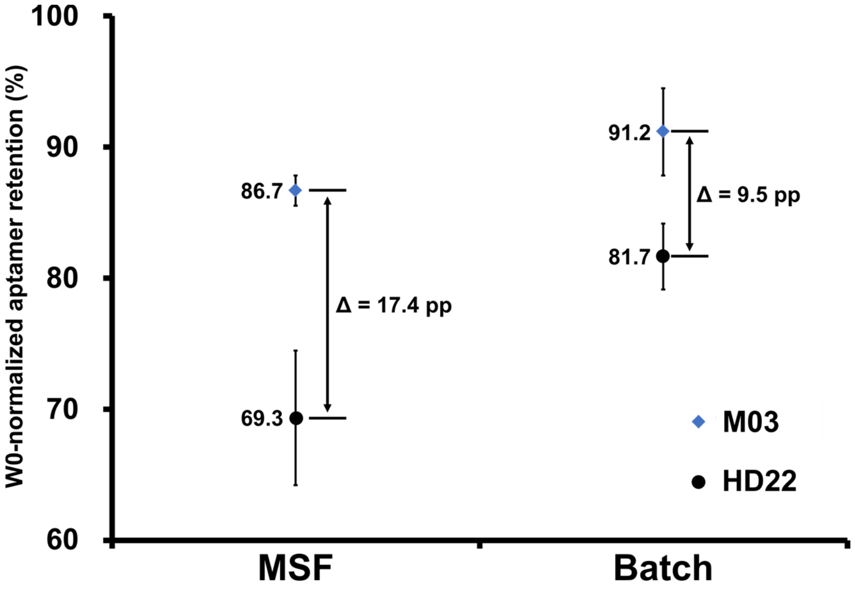

Figure S7. Comparison of centrifugal microspin filter and column-free magnetic separation washing formats. HD22 and M03 were evaluated using the centrifugal microspin filter (MSF) and column-free magnetic separation (Batch) formats. W0-normalized aptamer retention was calculated by normalizing the amount of aptamer recovered after 10 wash cycles (W10) to the directly measured W0 amount obtained by immediate elution without washing. Data are presented as the mean ± SD of three parallel process replicates (n = 3).

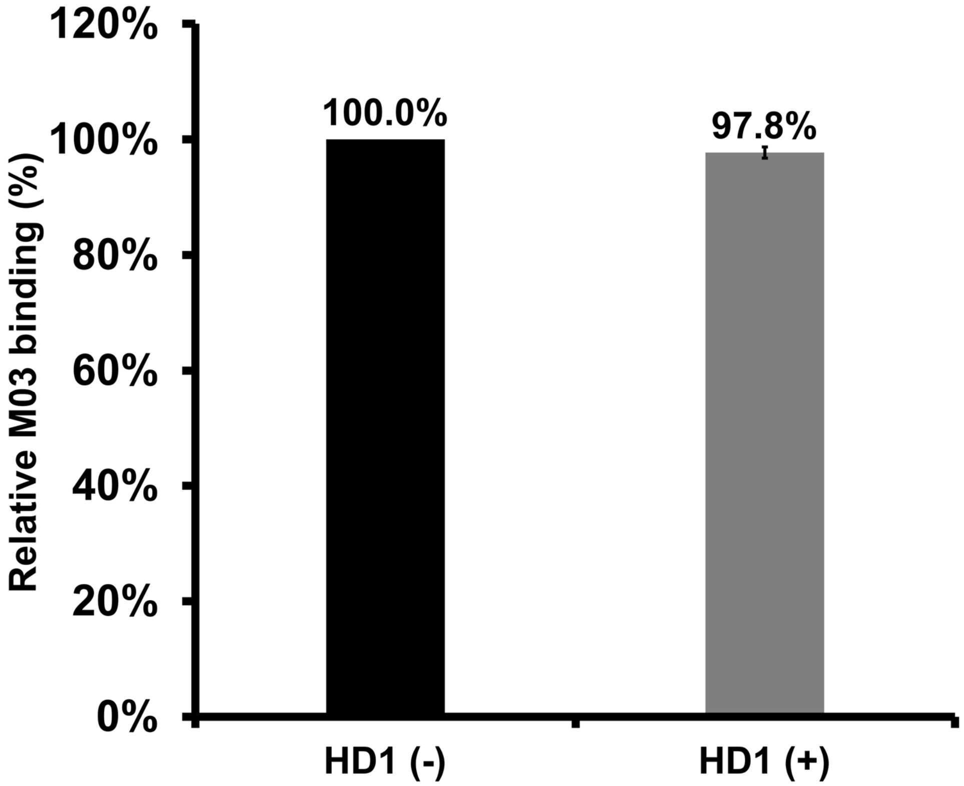

Figure S8. Effect of HD1 pre-binding on M03 binding to thrombin. Magnetic beads were incubated with 18.4 pmol of biotinylated thrombin for 1 h to immobilize the target. The thrombin-presenting beads were subsequently incubated with or without HD1 (200 pmol) for 1 h, followed by incubation with M03 (10 fmol) for 1 h. HD1 (−) and HD1 (+) indicate the absence and presence of HD1 pre-incubation, respectively. Bound M03 was quantified by qPCR and normalized to the mean value obtained in the HD1 (−) condition, which was defined as 100%. Data are presented as the mean ± SD of three parallel assay replicates (n = 3), with each sample quantified by qPCR in technical triplicate.

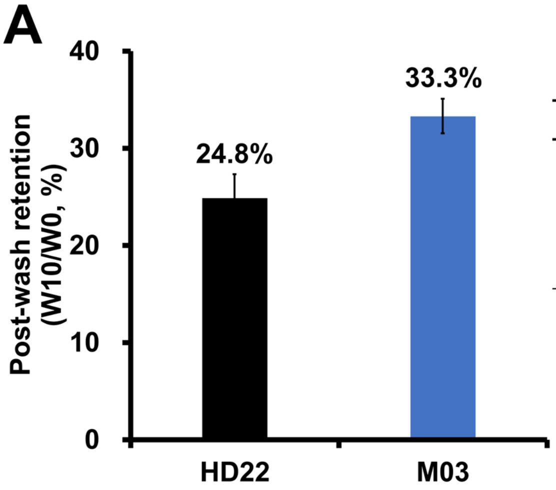

B **Negative controls**

Condition	qPCR signal
HD1 (−)	<LLOQ
Thrombin (−)	<LLOQ
Detection aptamer (−)	<LLOQ

Figure S9. Control experiments for the HD1-based thrombin sandwich detection assay. (**A**) Post-wash retention of HD22 and M03 under the complete sandwich condition. Streptavidin magnetic beads were incubated with 20 pmol of biotinylated HD1 for 1 h, followed by incubation with thrombin (50 nM in 50 μL) for 1 h and then with either HD22 or M03 (5 pmol in 50 μL) as the detection aptamer for 1 h. Bound detection aptamers were eluted either immediately without washing (W0) or after ten sequential washes with 500 μL of assay buffer, with each wash including a 2-min incubation (W10), and post-wash retention was calculated as W10/W0 × 100. (**B**) Component-omission controls performed in the absence of HD1, thrombin, or the detection aptamer. qPCR signals in all

omission controls were below the lower limit of quantification (<LLOQ). Data in (**A**) are presented as the mean ± SD of three parallel assay replicates (n = 3), with each sample quantified by qPCR in technical triplicate. LLOQ, lower limit of quantification.

Table S1. Oligonucleotide sequences used in this study. All sequences are shown in the 5′→3′ direction. In (**A**), common constant regions are shown in lowercase letters, whereas aptamer-derived regions are shown in uppercase letters. Full-length aptamer sequences correspond to the constructs experimentally evaluated in this study. For scrambled HD22, the constant regions were retained and the aptamer-derived region was randomly rearranged while preserving its nucleotide composition.

(A) Aptamer

ID	Full-length sequence (5'→3')	Length (nt)	Ref.
	**Thrombin aptamers**		
HD22	agatgcctgtcgagcatgctAGTCCGTGGTAGGG-CAGGTTGGGGTGACTgtagctaaactgctttgtcgacggg	74	[1]
M02	agcagcacagaggtcagatgAAGAGGGTGGAG-TGGTTGGCTTCACAATGGcctatgcgtgctaccgtgaa	70	[2]
M03	agcagcacagaggtcagatgGTGGTCGGGGTGGTGG-GATGAGGGTTCTGAcctatgcgtgctaccgtgaa	70	
M04	agcagcacagaggtcagatgGCGTGGTAGGG-CAGGTTGGGGTCCATGTTGcctatgcgtgc-taccgtgaa	70	
M07	agcagcacagaggtcagatgTTAGGGGTTGG-GAGGGTGGCTGACTAATGTAcctatgcgtgc-taccgtgaa	71	
M09	agcagcacagaggtcagatgGGGTT-GGATTGGGTGGCGGTGTGAACTATGcc-tatgcgtgctaccgtgaa	70	
M10	agcagcacagaggtcagatgAGCGGGGTT-GGGGGGGGTGGAGGAGCTCGTTcctatgcgtgc-taccgtgaa	71	
AYA1809001	tagggaagagaaggacatatgatGGTAGCG-TAAGGATGCGCAAGTTTAATT-GCCATATGCCATttgactagtacatgaccacttga	86	[3]
AYA1809002	tagggaagagaaggacatatgatGCGGTGTT-GCTGGGGTTGGGAGGTTGGAGGAA-GCAATCGCttgactagtacatgaccacttga	86	
AYA1809003	tagggaagagaaggacatatgatGTGTAG-GATGGGTGGGTGGGTCACATTTAAGA-TATCCTGGttgactagtacatgaccacttga	86	
AYA1809004	tagggaagagaaggacatatgatTCGTCAAGGTAC-GGTTGGGGGGGTGGGTGTTGACGTT-GAAttgactagtacatgaccacttga	86	
AYA1809005	tagggaagagaaggacatatgatGCG-GATGGATGGTGAGGTTGGGAGCTTTCATT-GGAACTAAttgactagtacatgaccacttga	86	
AYA1809007	tagggaagagaaggacatatga-tAGGGGCTCTAGGGTGGGTAGGATGGTGA-GACCGCAGCGTttgactagtacatgaccacttga	85	
	**Streptavidin aptamers**		
StrepApt1	ataccagcttattcaattGGGAACGCGTTATTGGG-TAACTTCCCacaatcgtaatcagttag	62	[4]
StrepApt2	ataccagcttattcaattGGGAATCGCCACCCGAC-GCAGGGTTTCCCacaatcgtaatcagttag	65	
StrepApt3	ataccagcttattcaattGGGAACGCATACGCCGCAG-TATTTCCCacaatcgtaatcagttag	63	
StrepApt4	ataccagcttattcaattGGGAACGCCCG-TATTGCTCGGTTCCCacaatcgtaatcagttag	62	

StrepApt5	ataccagcttattcaattGGGAACGCACCGATCG-CAGGTTTCCCacaatcgtaatcagttag	62	
St-2-1	ataccagcttattcaattATTGACCGCTGTGTGAC-GCAACACTCAATacaatcgtaatcagttag	65	[5]
**Control oligonucleotide**			
Scrambled HD22	agatgcctgtcgagcatgctGAGAGTGTGCGCTGAG-TGTGTGCGAGCGTgtagctaaactgctttgtcgacggg	74	-
**Capture aptamer**			
HD1	Biotin-TEG-GGTTGGTGTGGTTGG	15	[6]

(B) Primer

ID	Full-length sequence (5'→3')	Length (nt)
HD22_F	AGATGCCTGTCGAGCATGCT	20
HD22_R	CCCGTCGACAAAGCAGTTTAGCTAC	25
M_F	AGCAGCACAGAGGTCAGATG	20
M_R	TTCACGGTAGCACGCATAGG	20
AYA_F	TAGGGAAGAGAAGGACATATGAT	23
AYA_R	TCAAGTGGTCATGTACTAGTCAA	23
Strep_F	ATACCAGCTTATTCAATT	18
Strep_R	CTAACTGATTACGATTGT	18

Table S2. Summary of MSF–k_{off} assay metrics and SPR-derived kinetic parameters for thrombin aptamers.

Aptamer	I-NRC			W0-NRT			K_D (nM)	k_{on} (M^{-1} s^{-1})	k_{off} (s^{-1})
	Mean (%)	SD	CV (%)	Mean (%)	SD	CV (%)			
HD22	26.72	3.04	11.39	69.33	4.00	5.77	3.97	3.23×10^4	1.28×10^{-4}
M02	82.88	0.98	1.18	50.47	2.03	4.02	3.43	4.21×10^4	1.45×10^{-4}
M03	56.08	2.96	5.28	80.30	3.01	3.75	4.00	1.82×10^4	7.27×10^{-5}
M04	49.85	0.64	1.28	79.21	2.61	3.30	2.65	4.25×10^4	1.13×10^{-4}
M07	87.90	0.15	0.17	79.01	3.34	4.23	1.83	6.24×10^4	1.14×10^{-4}
M09	64.84	2.74	4.22	112.30	5.44	4.84	0.74	3.78×10^4	2.81×10^{-5}
M10	89.48	0.04	0.05	59.55	2.94	4.94	2.14	6.31×10^4	1.35×10^{-4}
AYA1809001	83.15	0.20	0.24	78.62	2.38	3.03	1.79	7.01×10^4	1.26×10^{-4}
AYA1809002	90.96	0.05	0.06	26.44	3.12	11.80	6.41	6.72×10^4	4.31×10^{-4}
AYA1809003	21.38	1.42	6.65	101.01	3.12	3.09	2.41	2.95×10^4	7.09×10^{-5}
AYA1809004	82.95	0.05	0.06	40.54	2.20	5.43	5.62	4.49×10^4	2.52×10^{-4}
AYA1809005	75.15	5.89	7.84	37.62	2.32	6.17	6.76	5.28×10^4	3.57×10^{-4}
AYA1809007	64.80	2.90	4.48	59.00	2.31	3.92	4.26	3.17×10^4	1.35×10^{-4}

Table S3. Correlation, partial correlation, and multivariable regression analyses of W0-NRT and SPR-derived equilibrium and kinetic parameters in the thrombin aptamer panel.

Analysis	Relationship	Statistic	*p* value	Additional information
Correlation	ln(W0-NRT) vs. K_D	Pearson's r = -0.865	0.00014	-
		Spearman's ρ = -0.731	0.0045	-
	ln(W0-NRT) vs. k_{on}	Pearson's r = -0.409	0.165	-
		Spearman's ρ = -0.473	0.103	-
	k_{off} vs. K_D	Pearson's r = 0.854	0.00020	-
	ln(k_{off}) vs. ln(K_D)	Pearson's r = 0.833	0.00041	-
Partial correlation	k_{off} vs. ln(W0-NRT), controlling for K_D	Partial r = -0.780	0.0028	-
	k_{off} vs. ln(W0-NRT), controlling for k_{on}	Partial r = -0.932	< 0.0001	-
	K_D vs. ln(W0-NRT), controlling for k_{off}	Partial r = -0.342	0.276	-
	k_{on} vs. ln(W0-NRT), controlling for k_{off}	Partial r = +0.166	0.607	-
Multivariable regression	ln(W0-NRT) ~ k_{off} + K_D	β = −0.754 (k_{off})	0.0028	R^2 = 0.901; VIF = 3.70
		β = −0.220 (K_D)	0.277	VIF = 3.70
	ln(W0-NRT) ~ k_{off} + k_{on}	β = −0.973 (k_{off})	< 0.0001	R^2 = 0.891; VIF = 1.31
		β = +0.063 (k_{on})	0.607	VIF = 1.31

W0-NRT values were natural-log transformed before analysis. β denotes the standardized regression coefficient. VIF, variance inflation factor.

Table S4. Comparison of thrombin aptamer pairs matched in one binding parameter while differing in other parameters.

Matched pair	Matching basis	K_D (nM)	k_{on} (M^{-1} s^{-1})	k_{off} (s^{-1})	W0-NRT (%)	*p* value
M10/AYA1809007	Similar k_{off}	2.14/4.26	6.31×10^4/3.17×10^4	1.35×10^{-4}/1.35×10^{-4}	59.55 ± 2.94/59.00 ± 2.31	0.81
M04/M07	Similar k_{off}	2.65/1.83	4.25×10^4/6.24×10^4	1.13×10^{-4}/1.14×10^{-4}	79.21 ± 2.61/79.01 ± 3.34	0.94
HD22/M03	Similar K_D	3.97/4.00	3.23×10^4/1.82×10^4	1.28×10^{-4}/7.27×10^{-5}	69.33 ± 4.00/80.30 ± 3.01	0.022
M02/M03	Similar K_D	3.43/4.00	4.21×10^4/1.82×10^4	1.45×10^{-4}/7.27×10^{-5}	50.47 ± 2.03/80.30 ± 3.01	0.0003
HD22/AYA1809001	Similar k_{off}	3.97/1.79	3.23×10^4/7.01×10^4	1.28×10^{-4}/1.26×10^{-4}	69.33 ± 4.00/78.62 ± 2.38	0.036

W0-NRT values are presented as mean ± SD of three parallel process replicates. *p* values were calculated using Welch's *t* test.

Table S5. Day-to-day reproducibility of W0-NRT and candidate ranking. W0-NRT was measured for M09, HD22, and AYA1809002 on two separate experimental days. Values are presented as the mean ± SD of three parallel process replicates for each day (n = 3). Candidate ranks were assigned

in descending order of W0-NRT, with rank 1 corresponding to the highest W0-NRT. Between-day CV was calculated from the Day 1 and Day 2 mean W0-NRT values.

Aptamer	Day 1 W0-NRT (%)	Day 2 W0-NRT (%)	Day 1 rank	Day 2 rank	Between-day CV (%)
M09	92.73 ± 3.44	99.76 ± 3.01	1	1	5.17
HD22	57.21 ± 3.20	65.32 ± 4.70	2	2	9.36
AYA1809002	26.48 ± 1.11	24.10 ± 2.01	3	3	6.66

Table S6. Summary of MSF–k_{off} assay metrics and literature-reported kinetic parameters for streptavidin aptamers.

Aptamer	I-NRC			W0-NRT			K_D* (nM)	k_{on}* (M^{-1} s^{-1})	k_{off}* (s^{-1})
	Mean (%)	SD	CV (%)	Mean (%)	SD	CV (%)			
StrepApt1	16.42	0.51	3.10	1.85	0.08	4.48	375	1.37×10^4	5.1×10^{-3}
StrepApt2	2.79	0.08	2.87	3.11	0.24	7.88	77	3.98×10^4	3.07×10^{-3}
StrepApt3	6.16	0.28	4.55	1.12	0.04	3.15	105	4.8×10^4	5.0×10^{-3}
StrepApt4	7.59	0.18	2.37	7.05	0.80	11.33	181	0.73×10^4	1.32×10^{-3}
StrepApt5	5.98	0.10	1.67	25.44	0.59	2.33	35	1.04×10^4	0.36×10^{-3}
St-2-1	24.59	1.35	5.49	5.61	0.19	3.43	79	3.2×10^4	2.5×10^{-3}

* The K_D, k_{on}, and k_{off} values are point estimates reported from SPR analyses in the original literature. The original publications also reported corresponding ± values for each kinetic parameter. For consistency with the thrombin aptamer table, only the point estimates are presented here. These literature-reported values were not obtained under experimental conditions matched to those used in the present study.

Table S7. Comparison of estimated assay-related costs for SPR and the MSF–k_{off} assay. (**A**) Overall comparison of the estimated assay-related cost per candidate and fixed costs for SPR and the MSF–k_{off} assay on matched analysis bases. Costs are shown for one MSF–k_{off} process replicate versus one SPR concentration series and for three MSF–k_{off} process replicates versus three SPR concentration series. The SPR cost was based on the publicly available price for full-service kinetic analysis provided by a single institution, whereas the MSF–k_{off} assay cost was calculated from the reagents and consumables required under the conditions used in this study. In the experiments reported here, the MSF–k_{off} assay was performed using three parallel process replicates, whereas SPR was performed using one concentration series per aptamer; the matched comparisons in (**A**) are provided specifically for cost comparison. The MSF–k_{off} assay cost does not include labor costs or the purchase or depreciation costs of the centrifuge, real-time PCR instrument, and magnetic stand. Therefore, the two values should be interpreted as illustrative local estimates rather than as an equivalent economic comparison. (**B**) Detailed breakdown of the estimated cost per candidate for the MSF–k_{off} assay using three parallel process replicates. Oligonucleotide synthesis costs were excluded because they were common to both methods.

(A) Overall cost comparison

Assay	Cost basis	Estimated cost per candidate (USD)	Fixed costs (USD)
SPR	One concentration series	260.00 [7]	Setup, 500; target preparation, 120 [7]
MSF–k_{off} assay	One process replicate	37.84	Not included
SPR	Three concentration series	780.00 [7]	Setup, 500; target preparation, 120 [7]

MSF–k_{off} assay	Three parallel process replicates	69.79	Not included

In the experiments performed in this study, the MSF–k_{off} assay was conducted using three parallel process replicates, whereas SPR was performed using one concentration series per aptamer. The one-versus-one and three-versus-three comparisons shown here are provided to allow cost comparison on matched replicate bases. Both MSF–k_{off} estimates include the aptamer-specific seven-point standard curve and the no-template control, which are required once per candidate irrespective of the number of process replicates; the three-replicate estimate is therefore lower than three times the one-replicate estimate. SPR setup and target-preparation fees are one-time fixed charges and are not included in the per-candidate estimates.

(B) Cost breakdown of the MSF–k_{off} assay

Cost component	Basis per candidate	Estimated cost (USD)
Biotinylated α-thrombin*	55.2 pmol	9.00
Streptavidin magnetic beads*	276 μg	6.00
Microspin filter units	3 units	12.83
DNA cleanup columns	3 columns	5.53
qPCR reagents	21 sample reactions + 24 standard/NTC reactions	36.43
**Total**		69.79

* For the MSF–k_{off} assay, one thrombin–bead preparation yielded 250 μL of suspension, and 50 μL was used per microspin filter unit. Thus, one preparation was sufficient for five filter units. For a candidate analyzed in three parallel process replicates, the bead and thrombin costs were calculated as three-fifths of one preparation, corresponding to 276 μg of beads and 55.2 pmol of thrombin. Preparations were used across candidates within the same experimental session rather than discarded after three filter units.

References

1. Tasset, D.M.; Kubik, M.F.; Steiner, W. Oligonucleotide Inhibitors of Human Thrombin That Bind Distinct Epitopes. *J. Mol. Biol.* **1997**, *272*, 688–698.
2. Wakui, K.; Yoshitomi, T.; Yamaguchi, A.; Tsuchida, M.; Saito, S.; Shibukawa, M.; Furusho, H.; Yoshimoto, K. Rapidly Neutralizable and Highly Anticoagulant Thrombin-Binding DNA Aptamer Discovered by MACE SELEX. *Mol. Ther. Nucleic Acids* **2019**, *16*, 348–359.
3. Ayass, M.A.; Griko, N.; Pashkov, V.; Tripathi, T.; Zhang, J.; Ramankutty Nair, R.; Okyay, T.; Zhu, K.; Abi-Mosleh, L. New High-Affinity Thrombin Aptamers for Advancing Coagulation Therapy: Balancing Thrombin Inhibition for Clot Prevention and Effective Bleeding Management with Antidote. *Cells* **2023**, *12*, 2230.
4. Ruigrok, V.J.B.; van Duijn, E.; Barendregt, A.; Dyer, K.; Tainer, J.A.; Stoltenburg, R.; Strehlitz, B.; Levisson, M.; Smidt, H.; van der Oost, J. Kinetic and Stoichiometric Characterisation of Streptavidin-Binding Aptamers. *ChemBioChem* **2012**, *13*, 829–836.
5. Bing, T.; Yang, X.; Mei, H.; Cao, Z.; Shangguan, D. Conservative Secondary Structure Motif of Streptavidin-Binding Aptamers Generated by Different Laboratories. *Bioorg. Med. Chem.* **2010**, *18*, 1798–1805.
6. Bock, L.C.; Griffin, L.C.; Latham, J.A.; Vermaas, E.H.; Toole, J.J. Selection of Single-Stranded DNA Molecules That Bind and Inhibit Human Thrombin. *Nature* **1992**, *355*, 564–566.
7. Scripps Research. Surface Plasmon Resonance. Available online: https://www.scripps.edu/science-and-medicine/cores-and-services/biophysics-and-biochemistry-core/spr/index.html (accessed on 31 August 2026). Archived version available online: https://perma.cc/6L3E-TDDB.
